# Supplementary material for: An investigation of discrepancies in outcome reporting and selective reporting bias in interrupted time series studies of health interventions: a methodological study
Source: BMC Public Health. 2026 May 13;26:2061. doi: 10.1186/s12889-026-27475-1 (PMC13339433; doi:10.1186/s12889-026-27475-1)
Supplement: Supplementary file 1 — Supplementary Material 1. [file 12889_2026_27475_MOESM1_ESM.pdf]

Check for updates

STUDY PROTOCOL

REVISED Investigation of reporting bias in interrupted time series (ITS) studies: a study protocol

[version 2; peer review: 2 approved, 1 approved with reservations]

Phi-Yen Nguyen[1], Simon Turner[1], Elizabeth Korevaar[1], Andrew Forbes[2], Matthew J. Page[1], Joanne E. McKenzie[1]

[1]Methods in Evidence Synthesis Unit, School of Public Health and Preventive Medicine, Monash University, St Kilda, Victoria, 3004, Australia
[2]Biostatistics Unit, School of Public Health and Preventive Medicine, Monash University, St Kilda, Victoria, 3004, Australia

v2 **First published:** 01 Mar 2024, **13**:153
https://doi.org/10.12688/f1000research.145927.1

Latest published: 04 Nov 2024, **13**:153
https://doi.org/10.12688/f1000research.145927.2

Abstract

Background

Interrupted time-series (ITS) studies are commonly used to examine the effects of interventions targeted at populations. Suppression of ITS studies or results within these studies, known as reporting bias, has the potential to bias the evidence-base on a particular topic, with potential consequences for healthcare decision-making. Therefore, we aim to determine whether there is evidence of reporting bias among ITS studies.

Methods

We will conduct a search for published protocols of ITS studies and reports of their results in PubMed, MEDLINE, and Embase up to December 31, 2022. We contact the authors of the ITS studies to seek information about their study, including submission status, data for unpublished results, and reasons for non-publication or non-reporting of certain outcomes. We will examine if there is evidence of publication bias by examining whether time-to-publication is influenced by the statistical significance of the study's results for the primary research question using Cox proportional hazards regression. We will examine whether there is evidence of discrepancies in outcomes by comparing those specified in the protocols with those in the reports of results, and we will examine whether the statistical significance of an outcome's result is associated with how completely that result is reported using multivariable logistic regression. Finally,

Open Peer Review

Approval Status ✔ ? ✔

	1	2	3
**version 2** (revision) 04 Nov 2024	✔ view		✔ view
**version 1** 01 Mar 2024	? view	? view	

1. **Jos Verbeek**, Coronel Institute of Occupational Health, Cochrane Work, Amsterdam, The Netherlands
2. **Qian Yu**, Jilin University, Changchun, China
3. **Alex Dahlen**, New York University, New York, USA

Any reports and responses or comments on the article can be found at the end of the article.

we will examine discrepancies between protocols and reports of results in the methods by examining the data collection processes, model characteristics, and statistical analysis methods. Discrepancies will be summarized using descriptive statistics.

Discussion

These findings will inform systematic reviewers and policymakers about the extent of reporting biases and may inform the development of mechanisms to reduce such biases.

Keywords
reporting bias, publication bias, selective reporting, outcome reporting, research methods, meta-research, interrupted time series, discrepancies in reporting

This article is included in the Research on Research, Policy & Culture gateway.

Corresponding author: Joanne E. McKenzie (joanne.mckenzie@monash.edu)

Author roles: Nguyen PY: Conceptualization, Data Curation, Formal Analysis, Investigation, Methodology, Writing – Original Draft Preparation; **Turner S**: Investigation, Supervision, Writing – Review & Editing; **Korevaar E**: Investigation, Writing – Review & Editing; **Forbes A**: Supervision, Writing – Review & Editing; **Page MJ**: Conceptualization, Investigation, Supervision, Writing – Review & Editing; **McKenzie JE**: Conceptualization, Funding Acquisition, Investigation, Methodology, Supervision, Writing – Review & Editing

Competing interests: No competing interests were disclosed.

Grant information: This work was supported by a Monash University's Departmental Scholarship for P-YN's Doctor of Philosophy (PhD) research program. MJP was supported by the Australian Research Council Discovery Early Career Researcher Award (DE200101618) during the conduct of this research and is currently part funded by the Research Support Package of Joanne E McKenzie's NHMRC Investigator Grant (GNT2009612) and a Monash University Future Leader Postdoctoral Fellowship (FLPF23-1069865460). ST and EK were funded by the Research Support Package of Joanne E McKenzie's NHMRC Investigator Grant (GNT2009612). JEM was supported by an NHMRC Investigator Grant (GNT2009612).
The funders had no role in study design, data collection and analysis, decision to publish, or preparation of the manuscript.

Copyright: © 2024 Nguyen PY *et al*. This is an open access article distributed under the terms of the Creative Commons Attribution License, which permits unrestricted use, distribution, and reproduction in any medium, provided the original work is properly cited.

How to cite this article: Nguyen PY, Turner S, Korevaar E *et al.* **Investigation of reporting bias in interrupted time series (ITS) studies: a study protocol [version 2; peer review: 2 approved, 1 approved with reservations]** F1000Research 2024, **13**:153 https://doi.org/10.12688/f1000research.145927.2

First published: 01 Mar 2024, **13**:153 https://doi.org/10.12688/f1000research.145927.1

REVISED **Amendments from Version 1**

We have made minor changes to the Abstract and the main text's Introduction, in response to reviewers' comments. We also elaborated on the Ethical considerations and Data availability sections. For more details, please refer to our response to the reviewers' reports.

Any further responses from the reviewers can be found at the end of the article

Introduction

An interrupted time series (ITS) study is a non-randomized design, commonly used to evaluate interventions targeted at populations (e.g., introduction of tobacco plain packaging laws on the number of calls to smoking cessation helplines)[1] when randomized trials are not practical, and in some circumstances, ethical. This design can be less susceptible to bias than other non-randomized designs, such as before-after designs.[2–5] The use of ITS designs in public health has been increasing with time,[6–8] but has gained particular prominence during the COVID-19 pandemic to evaluate the effectiveness of COVID-19 reduction strategies (e.g., lockdowns),[9,10] as well as their impact on non-COVID-19 conditions.[11–13] The design has primarily been used in high-income countries, and to a more limited extent in low- and middle-income countries.

In an ITS study, measurements of an outcome variable are often collected continuously over time and aggregated (using summary statistics such as counts or proportions) within, generally, regular time intervals (e.g., weekly, monthly) for analysis. The 'interruption' separates the time series into pre- and post-interruption segments. The underlying time trend in the pre-interruption segment can be estimated and used to predict what would have occurred in the post-interruption period had the interruption not occurred (referred to as the 'counterfactual'). Differences between the predicted trend and the observed post-interruption trend can be used to quantify the effects of the interruption, such as an immediate change at the time of interruption ('level change') or the change in slope between pre- and post-interruption ('slope change').[4,14,15]

Systematic reviews may be undertaken to collate and synthesize evidence on the effects of interventions. In reviews that examine the effects of policy interventions, ITS studies are likely to be eligible because evidence from randomized trials may be limited or unavailable. A key factor underpinning the validity of the findings from systematic reviews is the extent of reporting bias in the evidence-base.

Reporting bias can arise when there is suppression of entire studies (known as publication bias) or results within studies that are unfavorable to the study hypotheses (known as selective reporting bias) due to the nature of the results themselves (i.e., based on their direction, magnitude, or P-value).[16,17] Reporting bias has the potential to bias conclusions drawn in systematic reviews (with or without meta-analyses), with potential consequences for healthcare decision-making.[18–20] Unlike randomized trials, where prospective registration is required by ethics committees and journals,[21,22] and many trial registries exist, such requirements and registries do not exist for ITS studies.[23,24] As a result, the same drivers are not in place to prespecify outcomes and analysis plans, or to publish unfavorable results of ITS studies. Therefore, reporting bias may exist to a greater extent in ITS studies.

The extent of the reporting bias among ITS studies is unknown. We aim to determine the extent of reporting bias among ITS studies based on the following three objectives:

1. To examine whether the publication of ITS studies is influenced by their results (i.e., publication bias).

2. To examine (i) whether there are discrepancies between outcomes specified in protocols of ITS studies and reports of their results and (ii) whether there is evidence of selective result reporting among ITS studies.

3. To examine whether there are discrepancies in the reporting of methods between protocols and reports of ITS studies.

Protocol

Each of these objectives will be addressed in a separate analysis (Figure 1).

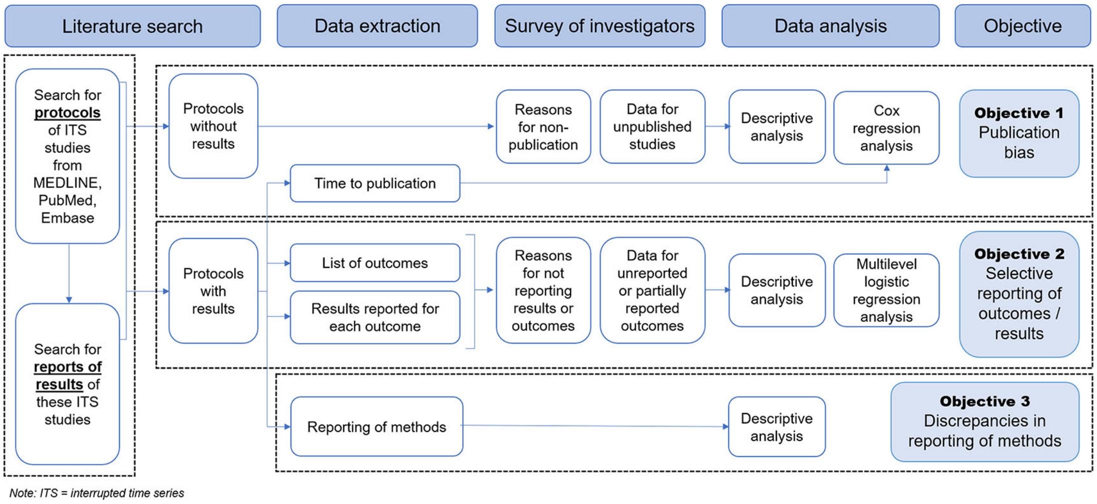

Figure 1. **Processes of literature search, data collection, survey and analysis.**

Creating a database of ITS study protocols and reports of their results

Identifying ITS study protocols

We will conduct a search for published protocols of ITS studies indexed in three bibliographic databases (PubMed, MEDLINE, and Embase via Ovid) and in the *JMIR Research Protocols*, each from inception to December 31, 2022. For bibliographic databases, we will use a sensitivity-maximizing search filter developed by our team for ITS studies.[25]

Eligible protocols include protocols and statistical analysis plans of ITS studies, including studies that plan to conduct ITS analysis alongside other planned analyses (e.g., qualitative analysis or cost-effectiveness modelling). For the purpose of our study, an ITS study is defined as one in which (a) there are at least two segments separated by a clearly defined interruption (i.e., an intervention or an exposure), (b) there are at least three data points for at least two of the segments, and (c) each data point represents a summary statistic (e.g., mean and rate) of individual observations collected from a group of individuals (e.g., within a country, state, hospital, or other unit) within a period of time (e.g., weekly or monthly). Both controlled and uncontrolled ITS studies will be considered eligible. We will only include ITS study protocols written in English.

One author (PYN) will screen all titles, abstracts, and potentially eligible full-text reports. A 10% random sample of full-text reports deemed ineligible and all full-text reports deemed eligible by the first author will be independently screened by the second author (JEM, ST, EK, or MJP). Discrepancies will be resolved through discussion between the authors or through team discussions.

Identifying corresponding reports of results

For each ITS study protocol meeting the inclusion criteria for our study, we will search for corresponding report(s) of the results using the following approaches: 1) searching in Ovid MEDLINE and Embase for the study's title and acronyms, trial registration number (if reported), and any co-author publications between the first author and last author; 2) checking for updates on registration sites, such as ClinicalTrials.gov; and 3) using forward citation searching tools, such as Web of Science's Cited Reference Search. For the purpose of this study, 'report(s) of the results' are defined as any peer-reviewed report that provides quantitative results for any outcome collected as part of the ITS. Other reports related to this study that present results for data not collected as part of the ITS will not be included. Methods paper utilising results from an ITS study will be excluded if not referenced in the protocol. If multiple eligible reports of results are found, we will include all of them. For each protocol, we will search for a report of the results only if at least 6 months have passed since the date of completion of data collection (as stated or implied in the ITS study protocol), or if not specified, after the date of publication of the protocol. We will not search for results if recruitment or data collection are confirmed to be ongoing at the time of the search.

Updating the search

We will begin by searching for protocols and reports of their results published up to December 31, 2022. This search will be updated once every six months until the time of analysis for each project, up to December 2023.

Data collection

Data extraction will be conducted using standardized extraction forms created in the Research Electronic Data Capture (REDCap)[26] hosted at Monash University. Information will be collected from the protocols and reports of the results (and all their supplementary files) and journal websites. Five authors (PYN, JEM, EK, MJP, and ST) will pilot the data extraction forms on five ITS studies to refine the items and achieve a shared understanding of the forms. One author (PYN) will extract the data for the remaining studies, and a second author (JEM, EK, MJP, or ST) will independently extract data for a random sample of 10% of the studies. For any items where we observed a high degree of inconsistency, we will undertake double data extraction for these items on a further randomly selected sample of studies. In addition, we will hold weekly meetings with all the authors to discuss any uncertainty arising during data extraction. Discrepancies will be resolved, and necessary amendments to the data extraction form will be made following these discussions. The data collection forms are summarized in Table 1.

Table 1. Summary of data collection.

Data from protocols	Data from report(s) of the results	Data from corresponding authors
**Basic information**		
• Details of corresponding authors<br>• Details of publication: date of submission, date of publication, name of journal<br>• Type of funding (government / not-for-profit/industry/ undisclosed/no funding)<br>• Elements constituting the primary interrupted time series (ITS) research question(s): populations/ setting, the interruptions, interruption groups and comparator groups<br>• Whether the study involves analyses other than ITS analysis (e.g. qualitative analysis)	• Details of publication: date of submission, date of publication, name of journal	• Whether data collection and analysis has been completed<br>• Whether a report of the ITS results has been submitted for publication<br>• Date of submission, rejection or publication of report of the results<br>• Reasons for not submitting results for publication<br>• Potential reasons for rejection of the report of results<br>• Name of journal
**Data collection process**		
• Eligibility criteria to select participants into the ITS<br>• Timing of data collection relative to publication of protocol: retrospective /prospective/both<br>• Start and end dates of the data to be collected<br>• Source of data (collected by investigating team/collected by an external party)<br>• Characteristics of all segments in the ITS: start and end date, and number of data points per segment	• Eligibility criteria to select participants into the ITS<br>• Timing of data collection relative to publication of protocol: retrospective /prospective/both<br>• Start and end dates of the data to be collected<br>• Source of data (collected by investigating team/collected by an external party)<br>• Characteristics of all segments in the ITS: start and end date, and number of data points per segment	
**Model characteristics**		
• Model structure (e.g. whether level change and/or slope change was modelled, whether the impact of the interruption was immediate or delayed, and how the interruption period was incorporated in analysis)	• Model structure (see details from 'Data from protocols')	

Table 1. *Continued*

Data from protocols	Data from report(s) of the results	Data from corresponding authors
**Model characteristics & statistical methods**		
• Estimation method (e.g. ordinary least squares, restricted maximum likelihood)<br>• Method of correcting for autocorrelation<br>• Method of correcting for seasonality<br>• Method of correcting for non-stationarity<br>• Method of handling outliers<br>• Covariates used for adjustment<br>• Presence of a control group<br>• Method of conducting intervention-control group comparison<br>• Subgroup analysis, if any	• Estimation method (e.g. ordinary least squares, restricted maximum likelihood)<br>• Method of correcting for autocorrelation<br>• Method of correcting for seasonality<br>• Method of correcting for non-stationarity<br>• Method of handling outliers<br>• Covariates used for adjustment<br>• Presence of a control group<br>• Method of conducting intervention-control group comparison<br>• Subgroup analysis, if any	
**Outcome-level information**		
• List of all planned outcomes<br>For each outcome:<br>• Description of outcome<br>• Whether higher value indicates benefit or harm<br>• Whether outcome was designated as primary or secondary outcome, or not specified<br>• Time intervals at which individual-level data were collected<br>• Time intervals at which data were aggregated for analysis<br>• Data type of individual-level observations (e.g. dichotomous, continuous)<br>• Data type of the summary statistic used to aggregate the individual-level observations (e.g. count, percentage)	• List of all reported outcomes<br>For each outcome:<br>• Description of outcome<br>• Whether higher value indicates benefit or harm<br>• Whether outcome was designated as primary or secondary outcome, or not specified<br>• Time intervals at which individual-level data were collected<br>• Time intervals at which data were aggregated for analysis<br>• Data type of individual-level observations (e.g. dichotomous, continuous)<br>• Data type of the summary statistic used to aggregate the individual-level observations (e.g. count, percentage)	For each outcome that was not reported or reported with discrepancy:<br>• Reasons for failure to report an outcome<br>• Reasons for reporting an outcome that was not specified in the protocol<br>• Reasons for inconsistency in labelling the primary status of outcome
**Result-level information**		
• List of all planned effect measures<br>For each effect measure<br>• Effect estimate<br>• Measure of precision (confidence interval and level (e.g. 95%), standard error)<br>• Exact P-value and threshold of statistical significance<br>• Direction of effect estimate (favouring interruption group/ comparator group)	• List of all reported effect measures<br>For each effect measure<br>• Effect estimate<br>• Measure of precision (95% confidence interval and level (e.g. 95%), standard error)<br>• Exact P-value and threshold of statistical significance<br>• Direction of effect estimate (favouring interruption group/ comparator group)	For each result that was not reported or not fully reported:<br>• Statistical significance of the effect estimate at 5% threshold<br>• Direction of effect estimate (favouring interruption group/comparator group)<br>• Reason for not reporting or not fully any the results

Study-level information

From each ITS study protocol, one author (PN) will identify the primary ITS research question(s), which will be used to determine which results are eligible for our assessment of publication and outcome/result reporting bias (see Box 1 for an example). In determining the primary ITS research question (or questions) from the protocol, we will only consider the population/setting, the interruption group(s), and the comparator group(s) elements of the research question and not the planned outcomes to be measured (note that we use the term 'group' to refer to interventions or exposures that occur in different time periods or segments). In a simple ITS with one interruption, there is only one comparison that can be made (between the pre- and post-interruption periods); for ITS with multiple interruptions, more than two comparisons are possible (Figure 2). Furthermore, the impact of interruption may be assessed in different populations. In reporting the

Box 1. Illustration of defining the primary research question(s) for the assessment of reporting biases.

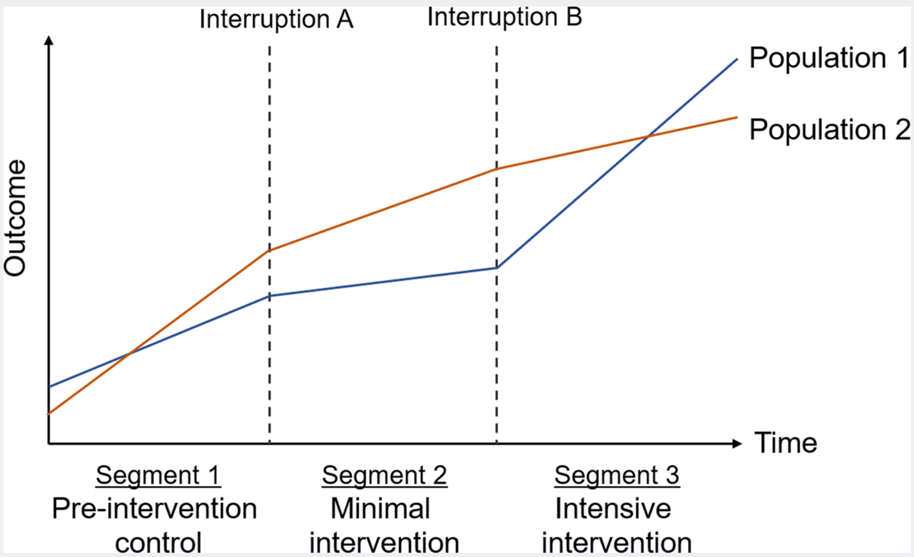

Suppose an interrupted time series (ITS) has three segments. The first segment is the pre-intervention control; the second is a minimal implementation of intervention A; and, the third is an intensive implementation of intervention A.

The intervention is evaluated in two populations (population 1 and 2).

If the authors stated "Our primary aim is to compare the intensive implementation A versus control", then for our assessment of publication and outcome/result reporting bias, we would consider any results pertaining only to this comparison, but for any population (since the population was not stated in the aim).

If the authors stated "Our primary aim is to examine the effect of intervention A", then for our assessment of publication and outcome/result reporting bias, we would consider any result pertaining to the comparisons 'minimal intervention A versus control', 'intensive intervention A versus control', 'intensive intervention A versus minimal intervention A'; and, for any population.

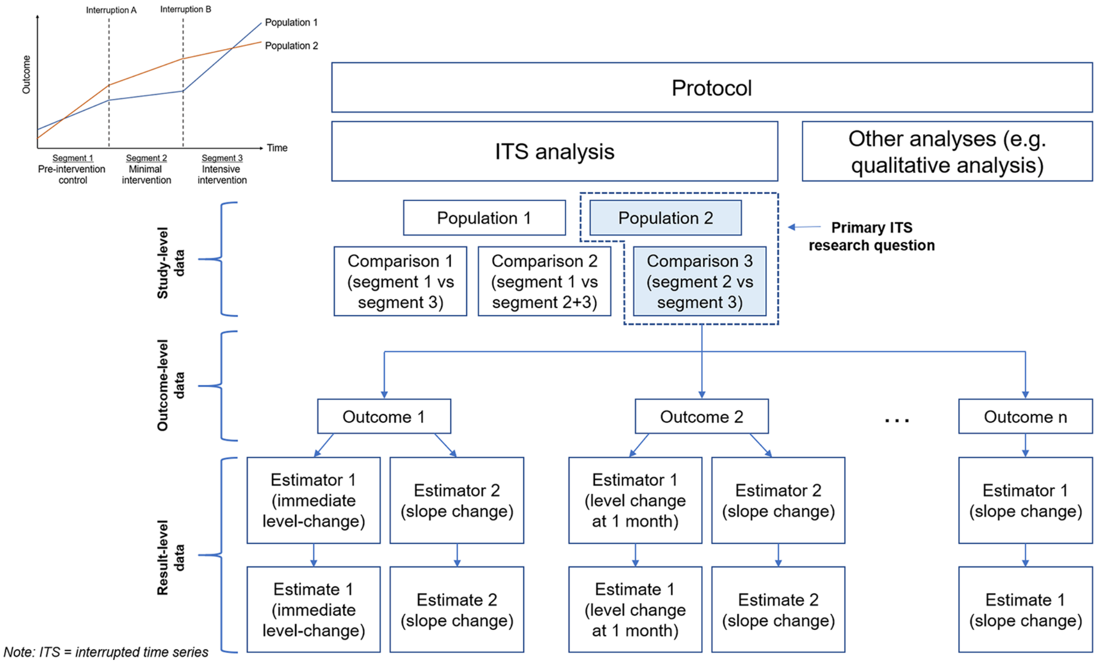

Figure 2. Structure of data to be collected for each ITS study.

research questions, authors may or may not be explicit about all elements or comparisons of primary interests. For the purpose of our study, we will use the reported primary question elements to determine which results are eligible for our assessment of publication and result reporting bias (see Box 1 for an example). The primary ITS question elements will be those that are reported by the authors as 'primary,' or the first reported in the protocol.

For each ITS study, we will record details of the methods, including the data collection process, model characteristics, and statistical analysis methods, as presented in both the protocol and report(s) of the results. We will record the eligibility criteria to select participants for the ITS, whether data collection is retrospective or prospective (or both), the start and end dates of the data to be collected, and whether the data were collected by the study authors or external to the study (e.g., collected as part of an administrative database). Data collection will be classified as retrospective if data were already available at the time of the protocol and as prospective if data will be collected during the study period. We will collect information to describe all time segments, including the start and end dates and the number of data points per segment. We will extract the model structure (if reported) or attempt to determine the model structure based on reported information (e.g., whether level change and/or slope change was modelled, whether the impact of the interruption was immediate or delayed, and how the interruption period was incorporated in the analysis). For statistical analysis methods, we will record the estimation method (e.g., ordinary least squares, restricted maximum likelihood), methods to deal with autocorrelation, seasonality, non-stationarity, and outliers; any adjustment for covariates; and use of control group and subgroup analyses.

In addition, we will record information related to the status of publication of the study results, as well as the journal name and date of publication for the protocol and all reports of the ITS results.

Outcome-level information
For all ITS outcomes addressing the primary research question(s), we will record the following details of the outcome, as presented in both the protocol and report(s) of the results: (a) the description of the outcome, (b) whether a higher value indicates benefit or harm, (c) whether the outcome was specified as a primary or secondary outcome, or neither; (d) the data type of the individual-level observations (e.g., dichotomous, continuous); and (e) the data type of the summary statistic used to aggregate the individual-level observations (e.g., count, percentage) within each period.

Result-level information
For all ITS results of the primary research question(s), we will record the effect measures reported (e.g. immediate level change, slope change), and (b) the available details about the results including: effect estimates, confidence intervals (along with level, e.g. 95%), exact P-values, statistical significance threshold (e.g. 5%), direction of the effect estimate (e.g. "favouring interruption group").

Correspondence with study authors
We will contact the corresponding authors to seek unpublished information about their studies when there are (i) no report(s) of the results or (ii) missing or incompletely reported results for the primary research questions (as defined in "Study-level information"). We will contact the authors using the email address provided in the ITS study protocol or report(s) of the results. We will send up to three reminders to each author at a minimum of two weeks apart in the case of non-response. If the corresponding author does not respond, we will attempt to contact the other authors.

Once the study authors provide informed consent to participate in the study, we will provide them with an electronic survey via Qualtrics. The survey will seek information on whether data collection and analysis were completed, whether the study has been submitted to a journal, date of submission, publication and/or rejection, reasons for not submitting and potential reasons for rejection, and the name of the corresponding journal.

For each ITS study, we will ask if the authors are willing to share information pertaining to the outcomes and results relevant to the primary ITS research question(s). We will ask the authors to share the reasons for not reporting an outcome, reporting an outcome that was not specified in the protocol, or for inconsistencies in labelling the primary status of the outcome. For each result that was not reported or not fully reported, we will ask the authors whether the result was statistically significant (at the 5% significance threshold) and whether the effect estimate favors the interruption or the comparator group.

The data received from the authors will be stored in a secure location. In reporting results from our analyses, individual studies or their results will not be identifiable.

Statistical analysis

Objective 1: Examining whether publication of ITS studies is influenced by their results (i.e. publication bias)

Only ITS protocols for which we have searched for report(s) of the results will be included in this analysis. Studies with ongoing recruitment or data collection, those confirmed to be abandoned, and those for which we have confirmed that the analysis has not been undertaken, will be excluded from our analysis.

We will calculate the percentage of protocols that do not have a report of ITS results and summarize the reasons why the results were not published. We define a 'report of ITS results' as a peer-reviewed report that includes results for *any* outcome pertaining to the primary ITS research question(s), as defined in "Study-level information". We define 'results' as quantitative results (e.g. effect estimate, 95% confidence interval, P-value), or qualitative statements about the statistical significance, P-value or direction of the effect estimate. Where data are available, each result will be classified as statistically significant (P-value <0.05 or, if absent, the 95% confidence interval not including the null) or not statistically significant (P-value ≥0.05 or, if absent, 95% confidence interval including the null). The direction of the result will be classified as "favouring interruption group" (i.e. showing beneficial effects or reducing harm) or "favouring comparator group".

We will undertake a multivariable Cox proportional hazards regression to determine whether a statistically significant effect estimate that favors the interruption group is associated with time to publication. Although statistical significance is not recommended for interpreting results, it is still widely used by researchers and journal editors to assess whether findings are potentially worth publishing[17,27] and, therefore, may influence time to publication.[28] Hazard ratios and 95% confidence intervals from the Cox regression analysis will be reported. *Time to publication of results* will be defined based on when the protocol is submitted relative to data collection. For studies where data collection was retrospective relative to the protocol submission, the time to publication will be from the protocol's submission date to the date of publication of the results. If the date of protocol submission has not been reported, we will substitute it with the date of protocol publication. For studies in which the protocol submission occurs prior to or during data collection, the time to publication will be from the last date of the data collection period to the date of publication of the results. If there is more than one report of the ITS results, we will calculate the time to publication for each report. Protocols for which results for the primary research question(s) are not available will be censored on the date of the last search for results. The following potential confounding factors will be included as covariates in the model: type of funding (government, not-for-profit, industry, undisclosed, or no funding), presence of prospective registration, and timing of data collection relative to the date of the protocol (retrospective, prospective, or both). We will adjust for potential correlations arising from clustering of results within studies, which might arise from studies having multiple outcomes, multiple comparisons, or multiple results (Figure 2).

Objective 2: Examining (i) whether there are discrepancies between outcomes specified in protocols and reports of ITS studies and (ii) whether there is evidence of selective result reporting among ITS studies

Studies with at least one ITS report will be eligible for this analysis. For the primary research question(s), as defined in "Study-level information", we will classify the study as having discrepancies in the reporting of outcomes if

- Any outcome specified in the protocol that was not mentioned in the report of the results.
- Any outcome reported in the report of the results was not pre-specified in the protocol.
- Any outcome was inconsistently labelled (e.g. being described as'primary' in the protocol but 'secondary' in the report of the results, or not being given a label in the protocol but being labelled as 'primary' or 'secondary' in the report of results). If an outcome is used in a power calculation, it will be considered the primary outcome.

For each effect estimate of each outcome, we classified the results using the approach proposed by Chan *et al.*[29] as follows:

- Fully reported – sufficient data is reported to include a result in a meta-analysis, that is, an effect estimate and a measure of precision (e.g., 95% confidence interval, standard error).
- partially reported – insufficient data are reported to include a result in a meta-analysis (e.g., an effect estimate is reported without any measure of precision); or

- qualitatively reported – only a statement about the statistical significance or the direction of the result (e.g. "there was no significant effect on road fatalities"), or only a P-value, is reported; or

- unreported – an outcome is mentioned in the protocol but no result is reported.

We will calculate the percentage of ITS studies that have discrepancies in the reporting of outcomes, the percentage of results that are fully reported, partially reported, qualitatively reported, or unreported, and summarize the reasons provided by the study authors for any discrepancy in reporting outcomes or failure to report any result. We will conduct a multilevel multivariable logistic regression to determine whether a statistically significant effect estimate that favors the interruption group is associated with the full reporting of the results. We willfit two models, one unadjusted and one adjusted for the following potential confounders: type of funding (government, not-for-profit, industry, undisclosed, no funding) and outcome status (primary/secondary/unspecified).[30] Both models will be adjusted for potential correlations arising from clustering of results within studies, which may arise from studies with multiple outcomes, multiple effect estimates for each outcome, and multiple comparisons (in series with more than two segments). In both models, calculated odds ratios and 95% confidence intervals will be reported.

Objective 3: Examining whether there are discrepancies in the reporting of methods between protocols and reports of ITS studies

Studies with at least one ITS report will be eligible for this analysis. If there are multiple results reports for the primary research question(s), we will select the report with the most detailed methods for comparison with the protocol. When this decision is not clear, we will determine the selected report via discussion between two authors (PN and EK/ST/MJP/JEM). For the primary research question(s), as defined in "Study-level information", we will compare the planned methods in the protocol and the primary report of the results to identify discrepancies in any aspect of the ITS methods, including the data collection process, model characteristics, and statistical analysis methods (see Table 1 for details). All discrepancies recorded will be reviewed by at least two statisticians (AF, EK, JEM, or ST) to judge whether the discrepancy was *important;* that is, whether the discrepancy could potentially change the result. A set of rules on what is considered an important discrepancy for each aspect of the methods will be determined via consensus among all the authors prior to data analysis. We will report the percentage of ITS studies that have any discrepancies and studies that have important discrepancies for each aspect of the methods. We will also summarize the rationales provided for the discrepancies, as reported by the study authors.

Discussion/conclusions

Selective non-publication of studies and selective non-reporting of results can bias conclusions drawn in systematic reviews (with or without meta-analyses).[18–20,31] Left-unaddressed, reporting biases in the ITS literature have the potential to lead to implementation of large-scale interventions that are at best, not effective, and at worst, harmful. Similar to other non-randomized studies, ITS literature is likely to be more prone to reporting biases than randomized trials because of a lack of mechanisms to encourage publication and reporting of all results, such as study registries, making registration a condition for publication, and guidelines for transparent reporting.[23,24,32] Furthermore, the analysis of ITS designs involves making many decisions, such as choice of the model structure, unit of time for aggregating observations, statistical methods, whether and how to adjust for autocorrelation, and other potential confounders, which can yield varied results depending on the decisions chosen.[4,15] Such multiplicity in analysis decisions provides an opportunity for study authors to report the most favorable results.

To our knowledge, this is the first study to systematically assess reporting biases among ITS studies across a range of topics and assess discrepancies in the reporting of methods between protocols and reports of their results. Knowledge of the prevalence of reporting biases will be useful for systematic reviewers and other stakeholders who rely on evidence from ITS studies and meta-analyses for decision-making. Furthermore, our results highlight the need for mechanisms to encourage complete reporting, such as the establishment of registries for non-randomized studies or planned analyses of existing datasets (e.g., administrative databases), along with incentives to register such studies and analyses. Moreover, based on our findings regarding the details of the methods most prone to discrepancies, future reporting checklists for ITS studies may incorporate recommendations for reporting these details.

Our study has some limitations. First, it is difficult to identify ITS studies at their inception. Many studies evaluating publication bias and selective reporting of results have focused on randomized trials, where the trials were identified by ethics committees.[33,34] We chose not to use ethics committees as a source for identifying ITS studies because ITS studies are sometimes undertaken without ethics approval being sought; thus, using this source would likely provide an incomplete sample of ITS studies. Instead, we will construct our sample from studies with a published protocol. However, using this sample may lead to underestimation of reporting biases, given that the presence of an *a priori* plan is associated

with a higher quality of design[35] and a lower likelihood of reporting biases.[36] Second, it is possible that we may miss report(s) of the results that are published in gray literature or not made public.

Ethical considerations

We will seek ethics approval from the Monash University Human Research Ethics Committee before contacting the study authors to clarify and seek missing information from their publications. A consent form will be sent to authors of the included ITS reports via email or via a Monash University approved survey provider (Qualtrics). Only data accompanied by signed consent forms will be included in the study. Findings from the quantitative analysis will be reported in aggregate to maintain study authors' confidentiality. Data will be stored on a Monash University secure server.

Data availability

Data that is extracted from the published ITS protocols and their associated reports will be deposited in a free-access data repository with a CC-BY license allowing reuse with attribution, and assigned a digital object identifier (DOI).

References

1. Young JM, Stacey I, Dobbins TA, *et al.*: **Association between tobacco plain packaging and Quitline calls: a population-based, interrupted time-series analysis.** *Med. J. Aust.* 2014 Jan 20 [cited 2023 Aug 25]; **200**(1): 29–32.
Publisher Full Text

2. Haber NA, Clarke-Deelder E, Salomon JA, *et al.*: **Impact Evaluation of Coronavirus Disease 2019 Policy: A Guide to Common Design Issues.** *Am. J. Epidemiol.* 2021 Nov 2; **190**(11): 2474–2486.
PubMed Abstract | Publisher Full Text | Free Full Text

3. Kontopantelis E, Doran T, Springate DA, *et al.*: **Regression based quasi-experimental approach when randomisation is not an option: interrupted time series analysis.** *BMJ.* 2015 Jun 9 [cited 2023 Jan 28]; **350**.
PubMed Abstract | Publisher Full Text | Free Full Text

4. Lopez Bernal J, Cummins S, Gasparrini A: **Interrupted time series regression for the evaluation of public health interventions: a tutorial.** *Int. J. Epidemiol.* 2017 Feb 1; **46**(1): 348–355.

5. Ramsay CR, Matowe L, Grilli R, *et al.*: **Interrupted time series designs in health technology assessment: Lessons from two systematic reviews of behavior change strategies.** *Int. J. Technol. Assess. Health Care.* 2003; **19**(4): 613–623.
PubMed Abstract | Publisher Full Text

6. Ewusie JE, Soobiah C, Blondal E, *et al.*: **Methods, Applications and Challenges in the Analysis of Interrupted Time Series Data: A Scoping Review.** *J. Multidiscip. Healthc.* 2020 May 13; **13**: 411–423.
PubMed Abstract | Publisher Full Text | Free Full Text

7. Hategeka C, Ruton H, Karamouzian M, *et al.*: **Use of interrupted time series methods in the evaluation of health system quality improvement interventions: a methodological systematic review.** *BMJ Glob. Health.* 2020 Oct; **5**(10): e003567.
PubMed Abstract | Publisher Full Text | Free Full Text

8. Hudson J, Fielding S, Ramsay CR: **Methodology and reporting characteristics of studies using interrupted time series design in healthcare.** *BMC Med. Res. Methodol.* 2019 Jul 4; **19**(1): 137.
Publisher Full Text

9. Navazi F, Yuan Y, Archer N: **The effect of the Ontario stay-at-home order on Covid-19 third wave infections including vaccination considerations: An interrupted time series analysis.** *PLoS One.* 2022 Apr 6; **17**(4): e0265549.
PubMed Abstract | Publisher Full Text | Free Full Text

10. Thayer WM, Hasan MZ, Sankhla P, *et al.*: **An interrupted time series analysis of the lockdown policies in India: a national-level analysis of COVID-19 incidence.** *Health Policy Plan.* 2021 Jun 3; **36**(5): 620–629.
PubMed Abstract | Publisher Full Text | Free Full Text

11. Mansfield KE, Mathur R, Tazare J, *et al.*: **Indirect acute effects of the COVID-19 pandemic on physical and mental health in the UK: a population-based study.** *Lancet Digit. Health.* 2021 Apr; **3**(4): e217–e230.
PubMed Abstract | Publisher Full Text | Free Full Text

12. Pirkis J, John A, Shin S, *et al.*: **Suicide trends in the early months of the COVID-19 pandemic: an interrupted time-series analysis of preliminary data from 21 countries.** *Lancet Psychiatry.* 2021 Jul; **8**(7): 579–588.
PubMed Abstract | Publisher Full Text | Free Full Text

13. Shah SA, Brophy S, Kennedy J, *et al.*: **Impact of first UK COVID-19 lockdown on hospital admissions: Interrupted time series study of 32 million people.** *eClinicalMedicine.* 2022 Jul 1 [cited 2023 Jun 20]; **49**.

14. Langan D, Higgins JPT, Jackson D, *et al.*: **A comparison of heterogeneity variance estimators in simulated random-effects meta-analyses.** *Res. Synth. Methods.* 2019 Mar 1; **10**(1): 83–98.
PubMed Abstract | Publisher Full Text

15. Turner SL, Karahalios A, Forbes AB, *et al.*: **Comparison of six statistical methods for interrupted time series studies: empirical evaluation of 190 published series.** *BMC Med. Res. Methodol.* 2021 Jun 26; **21**(1): 134.

16. Deeks JJ, Higgins JPT, Altman DG: **Chapter 10: Analysing data and undertaking meta-analyses.** *Cochrane Handbook for Systematic Reviews of Interventions. 6.3.*Cochrane Collaboration; 2022 [cited 2023 Feb 21].

17. Simmons JP, Nelson LD, Simonsohn U: **False-Positive Psychology: Undisclosed Flexibility in Data Collection and Analysis Allows Presenting Anything as Significant.** *Psychol. Sci.* 2011 Nov 1; **22**(11): 1359–1366.
Publisher Full Text

18. Kicinski M: **How does under-reporting of negative and inconclusive results affect the false-positive rate in meta-analysis? A simulation study.** *BMJ Open.* 2014 Aug 1; **4**(8): e004831.
PubMed Abstract | Publisher Full Text | Free Full Text

19. Smyth RMD, Kirkham JJ, Jacoby A, *et al.*: **Frequency and reasons for outcome reporting bias in clinical trials: interviews with trialists.** *BMJ.* 2011 Jan 6; **342**: c7153.
PubMed Abstract | Publisher Full Text | Free Full Text

20. Sutton AJ, Duval SJ, Tweedie RL, *et al.*: **Empirical assessment of effect of publication bias on meta-analyses.** *BMJ.* 2000 Jun 10; **320**(7249): 1574–1577.
PubMed Abstract | Publisher Full Text | Free Full Text

21. Chan AW, Pello A, Kitchen J, *et al.*: **Association of Trial Registration With Reporting of Primary Outcomes in Protocols and Publications.** *JAMA.* 2017 Nov 7; **318**(17): 1709–1711.
PubMed Abstract | Publisher Full Text | Free Full Text

22. Gopal AD, Wallach JD, Aminawung JA, *et al.*: **Adherence to the International Committee of Medical Journal Editors' (ICMJE) prospective registration policy and implications for outcome integrity: a cross-sectional analysis of trials published in high-impact specialty society journals.** *Trials.* 2018 Aug 23; **19**: 448.
PubMed Abstract | Publisher Full Text | Free Full Text

23. Quintana DS: **From pre-registration to publication: a non-technical primer for conducting a meta-analysis to synthesize correlational data.** *Front. Psychol.* 2015 [cited 2023 Jun 21]; **6**.
PubMed Abstract | Publisher Full Text | Free Full Text

24. Tanaka S, Tanaka S, Kawakami K: **Methodological issues in observational studies and non-randomized controlled trials in oncology in the era of big data.** *Jpn. J. Clin. Oncol.* 2015 Apr; **45**(4): 323–327.
PubMed Abstract | Publisher Full Text

25. Nguyen PY, McKenzie JE, Turner SL, *et al.*: **Development of a search filter to retrieve reports of interrupted time series studies from MEDLINE and PubMed.** *medRxiv.* 2023 [cited 2023 Sep 27]; 2023.09.14.23295594.

26. Harris PA, Taylor R, Thielke R, *et al.*: **Research electronic data capture (REDCap)-A metadata-driven methodology and workflow process for providing translational research informatics support.** *J. Biomed. Inform.* 2009 Apr; **42**(2): 377–381.
Publisher Full Text

27. Sterne JAC, Cox DR, Smith GD: **Sifting the evidence—what's wrong with significance tests? Another comment on the role of statistical methods.** *BMJ.* 2001 Jan 27; **322**(7280): 226–231.
PubMed Abstract | **Publisher Full Text** | **Free Full Text**

28. Hopewell S, Loudon K, Clarke MJ, *et al.*: **Publication bias in clinical trials due to statistical significance or direction of trial results.** *Cochrane Database Syst. Rev.* 2009 Jan 21; **2009**(1): MR000006.
PubMed Abstract | **Publisher Full Text**

29. Chan AW, Hróbjartsson A, Haahr MT, *et al.*: **Empirical evidence for selective reporting of outcomes in randomized trials: comparison of protocols to published articles.** *JAMA.* 2004 May 26; **291**(20): 2457–2465.
Publisher Full Text

30. Li G, Abbade LPF, Nwosu I, *et al.*: **A systematic review of comparisons between protocols or registrations and full reports in primary biomedical research.** *BMC Med. Res. Methodol.* 2018 Jan 11; **18**(1): 9.
PubMed Abstract | **Publisher Full Text** | **Free Full Text**

31. Page MJ, Higgins JP, Sterne JA: **Assessing risk of bias due to missing results in a synthesis.** *Cochrane Handbook for Systematic Reviews of Interventions.*John Wiley & Sons, Ltd; 2019 [cited 2023 Aug 25]; pp. 349–374.
Publisher Full Text

32. International Committee of Medical Journal Editors: **Recommendations for the Conduct, Reporting, Editing, and Publication of Scholarly Work in Medical Journals. Updated.** Jan 2024.

33. Dwan K, Gamble C, Williamson PR, *et al.*: **Systematic review of the empirical evidence of study publication bias and outcome reporting bias - an updated review.** *PLoS One.* 2013; **8**(7): e66844.
PubMed Abstract | **Publisher Full Text** | **Free Full Text**

34. Dwan K, Altman DG, Clarke M, *et al.*: **Evidence for the selective reporting of analyses and discrepancies in clinical trials: a systematic review of cohort studies of clinical trials.** *PLoS Med.* 2014 Jun; **11**(6): e1001666.
PubMed Abstract | **Publisher Full Text** | **Free Full Text**

35. Sideri S, Papageorgiou SN, Eliades T: **Registration in the international prospective register of systematic reviews (PROSPERO) of systematic review protocols was associated with increased review quality.** *J. Clin. Epidemiol.* 2018 Aug 100; **100**: 103–110.
Publisher Full Text

36. Chan AW, Hróbjartsson A: **Promoting public access to clinical trial protocols: challenges and recommendations.** *Trials.* 2018 Feb 17; **19**(1): 116.
PubMed Abstract | **Publisher Full Text** | **Free Full Text**

Open Peer Review

Current Peer Review Status: ✔ ? ✔

Version 2

Reviewer Report 10 December 2024

https://doi.org/10.5256/f1000research.173611.r345755

© **2024 Dahlen A.** This is an open access peer review report distributed under the terms of the Creative Commons Attribution License, which permits unrestricted use, distribution, and reproduction in any medium, provided the original work is properly cited.

✔ **Alex Dahlen**
New York University, New York, USA

This is a robust and clear study proposal to investigate an important topic. The authors have adequately addressed all the previous reviewer comments and I recommend indexing.
One small suggestion regarding the clause "that favors the interruption group". I suspect that for most studies, it will be relatively clear whether a result favors the interruption or control/baseline groups, but it might be the case that for some studies it could be hard to tell. As an example, a few years ago I wrote a paper with an ITS approach that examined the change in the rates of various medical procedures before and after covid lockdown. If there's a statistically significant drop in the rate of some procedure, does that "favor the interruption group"? It's kind of hard to tell. Maybe worth adding a sensitivity analysis where that clause is dropped and you look at results that are statistically significant *in either direction*. (I pre-registered that analysis with a post on Open Science Foundation, so it it won't be included in this analysis.)

Is the rationale for, and objectives of, the study clearly described?
Yes

Is the study design appropriate for the research question?
Yes

Are sufficient details of the methods provided to allow replication by others?
Yes

Are the datasets clearly presented in a useable and accessible format?
Not applicable

Competing Interests: No competing interests were disclosed.

Reviewer Expertise: public health study design, biostatistics

I confirm that I have read this submission and believe that I have an appropriate level of expertise to confirm that it is of an acceptable scientific standard.

Reviewer Report 09 December 2024

https://doi.org/10.5256/f1000research.173611.r337413

© 2024 Verbeek J. This is an open access peer review report distributed under the terms of the Creative Commons Attribution License, which permits unrestricted use, distribution, and reproduction in any medium, provided the original work is properly cited.

Jos Verbeek
Coronel Institute of Occupational Health, Cochrane Work, Amsterdam, The Netherlands

I am happy with the amendments and answers to my comments

Is the rationale for, and objectives of, the study clearly described?
Not applicable

Is the study design appropriate for the research question?
Not applicable

Are sufficient details of the methods provided to allow replication by others?
Not applicable

Are the datasets clearly presented in a useable and accessible format?
Not applicable

Competing Interests: No competing interests were disclosed.

Reviewer Expertise: environmental and occupational health, systematic reviews

I confirm that I have read this submission and believe that I have an appropriate level of expertise to confirm that it is of an acceptable scientific standard.

Version 1

Reviewer Report 10 September 2024

https://doi.org/10.5256/f1000research.159950.r268184

© 2024 Yu Q. This is an open access peer review report distributed under the terms of the Creative Commons Attribution License, which permits unrestricted use, distribution, and reproduction in any medium, provided the original work is properly cited.

Qian Yu
Jilin University, Changchun, China

I recommend the authors to address the aforementioned improvements. The study is well-positioned to contribute valuable insights into reporting biases in ITS studies, which is pivotal for enhancing the reliability of systematic reviews in healthcare research.
1. Explain why Cox proportional hazards regression and multivariable logistic regression were chosen as the main analysis tools.
2. Add cases of interrupted time series studies from different countries or regions to the background section, especially those related to reporting bias. This will help readers understand the global and prevalent nature of the issue. Compare studies internationally to analyze the potential causes and consequences of reporting bias across different countries or cultural backgrounds.
3. The study mentions seeking ethical approval and consent forms, but does not detail the procedures for protecting participant confidentiality and data security during the survey and data collection phases. Clarifying these aspects would strengthen the ethical considerations section.

Is the rationale for, and objectives of, the study clearly described?
Yes

Is the study design appropriate for the research question?
Yes

Are sufficient details of the methods provided to allow replication by others?
Yes

Are the datasets clearly presented in a useable and accessible format?
Yes

Competing Interests: No competing interests were disclosed.

Reviewer Expertise: Clinical pharmacy

I confirm that I have read this submission and believe that I have an appropriate level of expertise to confirm that it is of an acceptable scientific standard, however I have significant reservations, as outlined above.

Author Response 17 Oct 2024
Phoebe Nguyen

We would like to thank the reviewer for the thoughtful and helpful feedback. Please find our responses to each comment below.

FEEDBACK: I recommend the authors to address the aforementioned improvements. The study is well-positioned to contribute valuable insights into reporting biases in ITS studies, which is pivotal for enhancing the reliability of systematic reviews in healthcare research.

1. Explain why Cox proportional hazards regression and multivariable logistic regression were chosen as the main analysis tools.

RESPONSE: Under objective 1, we aim to investigate the impact of the direction and statistical significance of results on time to publication. Time to publication is time-to-event data, which means it incorporates two aspects: (i) whether the study was published or not, and if it was, (ii) the time until publication. Statistical methods that assume that the outcome is continuous can introduce bias; hence we are using a statistical method that was developed for time-to-event data – Cox proportional hazards regression.

Under objective 2, we aim to investigate the impact of the direction and statistical significance of results on how completely the result is reported. The outcome 'reporting status of the result' is a binary outcome (fully reported or not), and therefore logistic regression (with a binomial distribution and a logit link function) is an appropriate statistical method for this outcome type. We are using multivariable regression because we wish to adjust for potential confounding variables.

FEEDBACK: Add cases of interrupted time series studies from different countries or regions to the background section, especially those related to reporting bias. This will help readers understand the global and prevalent nature of the issue. Compare studies internationally to analyze the potential causes and consequences of reporting bias across different countries or cultural backgrounds.

RESPONSE: The ITS studies we cite in the Introduction were set in multiple countries (#9-12): Canada (#9), India (#10) and UK (#11); one country investigated 21 countries across multiple regions and income levels (#12). We have also added the following sentence to the Introduction which reflects the findings from methods reviews examining the characteristics of ITS studies: "The design has primarily been used in high-income countries, and to a more limited extent in low- and middle-income countries."
Regarding the reviewer's suggestion to provide information in the Introduction on the prevalence of reporting bias in ITS studies, and on the potential causes and consequences of reporting bias across different countries and cultural backgrounds, we are unaware of any studies that have examined reporting bias in the ITS literature; hence the reason for undertaking the present study.

FEEDBACK: The study mentions seeking ethical approval and consent forms, but does not detail the procedures for protecting participant confidentiality and data security during the survey and data collection phases. Clarifying these aspects would strengthen the ethical considerations section.

RESPONSE: We now provide more information about confidentiality and data security under the 'Ethical considerations' Section: "We will seek ethics approval from the Monash University Human Research Ethics Committee before contacting the study authors to clarify and seek missing information from their publications. A consent form will be sent to authors of the included ITS reports via email or via a Monash University approved survey provider (Qualtrics). Only data accompanied by signed consent forms will be included in the study. Findings from the quantitative analysis will be reported in aggregate to maintain

study authors' confidentiality. Data will be stored on a Monash University secure server."

We have also clarified that only data that is publicly available from the ITS protocols and their associated reports will be made available in the 'Data availability' Section: "Data that is extracted from the published ITS protocols and their associated reports will be deposited in a free-access data repository with a CC-BY license allowing reuse with attribution, and assigned a digital object identifier (DOI)."

Competing Interests: We declare no competing interests.

Reviewer Report 19 June 2024

https://doi.org/10.5256/f1000research.159950.r289038

© **2024 Verbeek J.** This is an open access peer review report distributed under the terms of the Creative Commons Attribution License, which permits unrestricted use, distribution, and reproduction in any medium, provided the original work is properly cited.

?

Jos Verbeek
Coronel Institute of Occupational Health, Cochrane Work, Amsterdam, The Netherlands

This is a protocol for a study of a cohort of protocols for interrupted timeseries studies (ITS) to evaluate if there is outcome reporting bias in the final study reports or whether there is publication bias due to the study report not being published.

General comment: I agree with the authors that ITS studies are a valuable design to evaluate higher level policy or legal interventions where experimental studies are difficult to perform because of the interventions are applied to a large group of persons. The main prerequisite for an ITS study is that the outcome data are routinely collected. Therefore, I wouldn't immediately consider outcome reporting bias as one of the biggest threats to their validity. The authors don't have much choice for the outcome, either it is available or not. Nevertheless, it might be good to evaluate this.

More specific comments:

- I was a bit confused if this was now about publication bias in systematic reviews (SR) or about outcome reporting in studies. The authors write: 'However, the suppression of ITS studies or results within these studies (known as reporting bias) has the potential to bias conclusions drawn in such systematic reviews, with potential consequences for healthcare decision-making. Therefore, we aim to determine whether there is evidence of reporting bias among ITS studies.' The suppression of ITS studies must refer to SRs but as such it sounds as if the authors of the SR would actively not include studies based on their results. I think this does not make sense. I think it would help if it were clear that both publication bias and outcome reporting bias will be studied from the perspective of the study authors. Outcome reporting bias can also occur in a SR but that will not be studied here. The final objective is of course to prevent distortion of the results in the evidence base in a SR but

that will not be studied here.
- I think it would be helpful to better define reporting bias and how it will be studied at the very start of the protocol. Now the authors write: 'A key factor underpinning the validity of the findings from systematic reviews is the extent to which the included studies (and their results) are representative of missing studies, a concept known as reporting bias.' I think this is not totally correct and is much better described later on where they say: 'Reporting bias can arise when there is suppression of entire studies (known as publication bias) or results within studies that are unfavorable to the study hypotheses (known as selective reporting bias) due to the nature of the results themselves (i.e., based on their direction, magnitude, or P-value)
- I think the main issue with this protocol is that there will be no ITS-protocols. It is like the authors write: 'Unlike randomized trials, where prospective registration is required by ethics committees and journals,21,22 and many trial registries exist, such requirements and registries do not exist for ITS studies.' This sentence undermines the whole study. If there are no protocols, there is no study or it will be an open-door result that there are no protocols. Can the authors please confirm that they know that there is a sufficient number of protocols available to do this study?
- It is laudable that the authors define their data clearly. However, I think this one on the direction of the effect estimate (e.g. "favouring interruption group") is not correct. Later the write similarly: The direction of the result will be classified as "favouring interruption group" (i.e. showing beneficial effects or reducing harm) or "favouring comparator group". For most ITS there is no comparison group and that is the very reason that the ITS design was chosen. The direction should be defined as 'favouring the intervention time-period' of 'favouring the pre-intervention time-period'. Also when there is a comparison group, it is usual to take the difference in the outcomes between the intervention and the comparison group and analyze if the effect size is different between the pre and post intervention period.
- If I understand correctly what the authors want to do, they will assemble a cohort of protocols and the protocol will be the study unit. Where it is said: 'Only ITS studies for which we have searched for report(s) of the results will be included in this analysis.', I assume that is meant 'Only ITS protocols for which..
- I got confused by the following statements about how to classify the study as having discrepancies in the reporting of outcomes if
-
    - Any outcome specified in the protocol was mentioned in the report of the results.
    - An outcome reported in the report of the results was not pre-specified in the protocol.

I think the first one does not show a discrepancy and should be deleted.

Is the rationale for, and objectives of, the study clearly described?

Yes

Is the study design appropriate for the research question?

Partly

Are sufficient details of the methods provided to allow replication by others?

Yes

Are the datasets clearly presented in a useable and accessible format?

Not applicable

Competing Interests: No competing interests were disclosed.

Reviewer Expertise: environmental and occupational health, systematic reviews

I confirm that I have read this submission and believe that I have an appropriate level of expertise to confirm that it is of an acceptable scientific standard, however I have significant reservations, as outlined above.

Author Response 17 Oct 2024

Phoebe Nguyen

We would like to thank the reviewer for the thoughtful and helpful feedback. Please find our responses to each comment below.

FEEDBACK: This is a protocol for a study of a cohort of protocols for interrupted timeseries studies (ITS) to evaluate if there is outcome reporting bias in the final study reports or whether there is publication bias due to the study report not being published.
General comment: I agree with the authors that ITS studies are a valuable design to evaluate higher level policy or legal interventions where experimental studies are difficult to perform because of the interventions are applied to a large group of persons. The main prerequisite for an ITS study is that the outcome data are routinely collected. Therefore, I wouldn't immediately consider outcome reporting bias as one of the biggest threats to their validity. The authors don't have much choice for the outcome, either it is available or not. Nevertheless, it might be good to evaluate this.

RESPONSE: We agree with the reviewer that many ITS studies use routinely collected data to examine the impacts of the interruption. However, in our methodological reviews examining design characteristics of ITS studies, we have found this is not always the case (Korevaar et al, 2022; Turner et al, 2020). Furthermore, even where routinely collected data is used, there may be multiple outcomes available that could be used to assess the impact of the interruption, providing opportunities for selective reporting of outcomes. For example, in ITS studies examining the effects of antimicrobial stewardship programs to reduce inappropriate antimicrobial prescribing, routine data is collected on prescribing of all antimicrobial agents (e.g. amikacin, azithromycin, cefepime, ceftazidime, ceftriaxone), of which some, or all, could be used to evaluate the impact of the intervention.

FEEDBACK: I was a bit confused if this was now about publication bias in systematic reviews (SR) or about outcome reporting in studies. The authors write: 'However, the suppression of ITS studies or results within these studies (known as reporting bias) has the potential to bias conclusions drawn in such systematic reviews, with potential consequences for healthcare decision-making. Therefore, we aim to determine whether there is evidence of reporting bias among ITS studies.' The suppression of ITS studies must refer to SRs but as such it sounds as if the authors of the SR would actively not include studies based on their results. I think this does not make sense. I think it would help if it were clear that both publication

bias and outcome reporting bias will be studied from the perspective of the study authors. Outcome reporting bias can also occur in a SR but that will not be studied here. The final objective is of course to prevent distortion of the results in the evidence base in a SR but that will not be studied here.

RESPONSE: We have amended the background of the abstract to make it clear that our focus is on reporting bias of individual ITS studies. The text now reads:
“Interrupted time-series (ITS) studies are commonly used to examine the effects of interventions targeted at populations. Suppression of ITS studies or results within these studies, known as reporting bias, has the potential to bias the evidence-base on a particular topic, with potential consequences for healthcare decision-making. Therefore, we aim to determine whether there is evidence of reporting bias among ITS studies.”

FEEDBACK: I think it would be helpful to better define reporting bias and how it will be studied at the very start of the protocol. Now the authors write: ‘A key factor underpinning the validity of the findings from systematic reviews is the extent to which the included studies (and their results) are representative of missing studies, a concept known as reporting bias.’ I think this is not totally correct and is much better described later on where they say: ‘Reporting bias can arise when there is suppression of entire studies (known as publication bias) or results within studies that are unfavorable to the study hypotheses (known as selective reporting bias) due to the nature of the results themselves (i.e., based on their direction, magnitude, or P-value)

RESPONSE: We have amended the text as follows:
“Systematic reviews may be undertaken to collate and synthesize evidence on the effects of interventions. In reviews that examine the effects of policy interventions, ITS studies are likely to be eligible because evidence from randomized trials may be limited or unavailable. A key factor underpinning the validity of the findings from systematic reviews is the extent of reporting bias in the evidence-base. Reporting bias can arise when there is suppression of entire studies […]”.

FEEDBACK: I think the main issue with this protocol is that there will be no ITS-protocols. It is like the authors write: ‘Unlike randomized trials, where prospective registration is required by ethics committees and journals,21,22 and many trial registries exist, such requirements and registries do not exist for ITS studies.’ This sentence undermines the whole study. If there are no protocols, there is no study or it will be an open-door result that there are no protocols. Can the authors please confirm that they know that there is a sufficient number of protocols available to do this study?

RESPONSE: Since submitting the protocol, we have completed the search and eligibility screening from which we have identified 158 protocols for ITS studies.

FEEDBACK: It is laudable that the authors define their data clearly. However, I think this one on the direction of the effect estimate (e.g. “favouring interruption group”) is not correct.

Later the write similarly: The direction of the result will be classified as “favouring interruption group” (i.e. showing beneficial effects or reducing harm) or “favouring comparator group”. For most ITS there is no comparison group and that is the very reason that the ITS design was chosen. The direction should be defined as ‘favouring the intervention time-period’ of ‘favouring the pre-intervention time-period’. Also when there is a comparison group, it is usual to take the difference in the outcomes between the intervention and the comparison group and analyze if the effect size is different between the pre and post intervention period.

RESPONSE: We have used the term ‘group’ to refer to interventions or exposures that occur in different time periods (segments). This is similar to the use of ‘group’ in controlled trials, except that in trials, groups are formed by assigning units, whereas in ITS studies, they are defined by time. To make this clearer, we have added the following sentence in Section 2.1: “Note that we use the term ‘group’ to refer to interventions or exposures that occur in different time periods (segments).”

FEEDBACK: If I understand correctly what the authors want to do, they will assemble a cohort of protocols and the protocol will be the study unit. Where it is said: ‘Only ITS studies for which we have searched for report(s) of the results will be included in this analysis.’, I assume that is meant ‘Only ITS protocols for which..

RESPONSE: The reviewer has understood correctly. We have amended the sentence to “Only ITS *protocols* for which…”.

FEEDBACK: I got confused by the following statements about how to classify the study as having discrepancies in the reporting of outcomes if

- Any outcome specified in the protocol was mentioned in the report of the results.
- An outcome reported in the report of the results was not pre-specified in the protocol.

I think the first one does not show a discrepancy and should be deleted.

RESPONSE: We have amended the sentence to “Any outcome specified in the protocol *that* was *not* mentioned in the report of the results”.

Competing Interests: We declare no competing interests.

The benefits of publishing with F1000Research:

- Your article is published within days, with no editorial bias
- You can publish traditional articles, null/negative results, case reports, data notes and more
- The peer review process is transparent and collaborative
- Your article is indexed in PubMed after passing peer review
- Dedicated customer support at every stage

For pre-submission enquiries, contact research@f1000.com

F1000Research
