# Supplementary material for: An investigation of discrepancies in outcome reporting and selective reporting bias in interrupted time series studies of health interventions: a methodological study
Source: BMC Public Health. 2026 May 13;26:2061. doi: 10.1186/s12889-026-27475-1 (PMC13339433; doi:10.1186/s12889-026-27475-1)
Supplement: Supplementary file 2 — Supplementary Material 2. [file 12889_2026_27475_MOESM2_ESM.docx]

# **S2. Deviations from protocol**

| **Original plan** | **Revised plan** | **Reason for modification** |
| --- | --- | --- |
| We planned to search for published protocols of interrupted time series (ITS) studies indexed in three bibliographic databases (PubMed, MEDLINE, and Embase via Ovid) and in the JMIR Research Protocols. | We included additional bibliographic databases (e.g. CINAHL, CENTRAL, Web of Science) and other types of sources (e.g., open access repositories, grey literature databases, trial registries), adding to a total of 22 sources. | This maximises the likelihood of capturing all potential ITS study protocols, since the search filter may not capture protocols that do not have the term “interrupted time series” in their titles and abstracts. |
| We planned to search for corresponding report(s) of the results in Ovid MEDLINE and Embase, trial registration sites, and using forward citation searching tools, such as Web of Science’s Cited Reference Search. | We included three more sources for searching: PubMed, ConnectedPapers.com and Google Scholar. | This maximises the likelihood of capturing all potential ITS results reports. |
| We planned to request information from the corresponding authors of the ITS studies on whether the unpublished results were statistically significant and favouring the intervention, or not. | We did not contact the corresponding authors, except to enquire on the publication status of three studies. | Lack of time and resources. |
| We did not have a working definition for an outcome. | We developed a framework to define an outcome based on five elements (domain, specific measure, time points, data types of individual measurements, and data types at aggregation). | We needed a structured approach to distinguish between potentially similar outcomes, and to match an outcome in the protocol with a corresponding outcome in the results report. |
| We assumed each outcome in the protocol was matched to only one outcome in the results report. | Each outcome in the protocol was either matched to a single outcome (a one-to-one match) or multiple outcomes (a one-to-many match) in the results report. | We had difficulty matching outcomes that were vaguely or broadly defined in the protocol, which potentially could be matched to several outcomes in the results report. Without sufficient information, we could not ensure exact one-to-one matching would be accurate. |
| We planned to include all outcomes in the assessment of discrepancies. | Outcomes with one-to-many matching were excluded from assessment of discrepancies. | Since we could not determine the exact one-to-one matching, we could not ensure the assessment of discrepancy in reporting would be accurate for these outcomes. |
| The covariate “funders” was planned to be classified as: government, not-for-profit, industry, undisclosed, no funding. | We classified “funders” as industry funders, non-industry funders (government, education institutions and all not-for-profit entities), no funder (as confirmed by authors) or no funding information. | We merged the categories “government” and “not-for-profit” funders as we found many not-for-profit funders are typically independent research institutions with significant government funding, and their influence on publication of research is similar enough to be grouped into one category. |
| We did not plan for sensitivity analyses. | We conducted a sensitivity analysis, where discrepancies with justification were classified as not a discrepancy. | We wanted to investigate the extent of “true” discrepancies i.e. discrepancies that were not justified or explained by the authors. |

# **S3. Data extraction form**

| **Question** | **Options** |
| --- | --- |
| **Information from protocols** | |
| **Title of protocol** | text |
| **What is the name of the publishing journal?** | text |
| **Does the protocol describe other planned analyses in addition to the ITS analysis (e.g. interviews, a pre-post analysis, cost-effectiveness study)**  The protocol often refers to these as multiple objectives, aims, sub-studies, or work packages (WPs). | multiple-choice   \| 1 \| Yes \| \| --- \| --- \| \| 0 \| No \| |
| **What is the source of funding?**  In-kind materials are also considered funding, and should be described in subsequent questions about the role of the funder. | checkbox   \| 99 \| Cannot be determined \| \| --- \| --- \| \| 0 \| No funding \| \| 1 \| Non-industry (non-profit, academic, government) \| \| 2 \| Industry \| \| 999 \| Other [elaborate] \| |
| **Briefly describe the intervention(s) or exposure(s) that constitute the interruption points.** | text |
| **What is the nature of interruption?**   - Natural events: e.g. disease outbreaks, weather-related or geological events (floods, earthquakes) - Unplanned human-made events: unintended or unforeseen human-driven events e.g. economic recession, environmental disasters, industrial accidents - Practice change in a clinical setting: a new or modified clinical practice, treatment, care model/pathway, etc. This also includes strategies to facilitate such implementation (e.g. facilitators, education and training). - Health system interventions: interventions involving systemic changes at multiple levels e.g. health system strengthening, workforce changes, complex interventions involving multiple stakeholders - Policy and regulatory changes: modifications in laws, national guidelines, or health system regulations e.g. taxation, lockdowns, national vaccination programmes - Social and economic interventions: initiatives that extend beyond healthcare, addressing broader social and economic determinants of well-being e.g. cash grants, microfinancing, health insurance model - Environmental interventions: modifications to the living and natural environment to influence public health e.g. mosquito control programs, urban planning | multiple-choice   \| 99 \| Cannot be determined \| \| --- \| --- \| \| 1 \| Exposure: Natural events \| \| 2 \| Exposure: Unplanned human-made events \| \| 3 \| Intervention: Practice change in a clinical setting \| \| 4 \| Intervention: Health system interventions \| \| 5 \| Intervention: Policy & regulatory changes \| \| 6 \| Intervention: Social & economic interventions \| \| 7 \| Intervention: Environmental interventions \| \| 999 \| Other [elaborate] \| |
| **At which level will the interruption occur, or be delivered / implemented?**   - Individual: The intervention is implemented in specific individuals without the intention to represent a geographical area. - Unit-based or institutions: The intervention is implemented in one or more specific institutions, hospitals or departments within a hospital. - Regional: The intervention is implemented in an entire district, state, province or region, or in a group of institutions purposely sampled to represent a region. - National: The intervention is implemented in multiple districts/regions in a country, or the entire country, or in a group of institutions purposely sampled to represent a country. - Multinational: The intervention is implemented in multiple countries. | multiple-choice   \| 99 \| Cannot be determined \| \| --- \| --- \| \| 1 \| Individual \| \| 2 \| Unit-based or institutional \| \| 3 \| Regional \| \| 4 \| National \| \| 5 \| Multinational \| |
| **At which level will the effect of the interruption be assessed in this study?**   - Individual: The intervention is implemented in specific individuals without the intention to represent a geographical area. - Unit-based or institutions: The intervention is implemented in one or more specific institutions, hospitals or departments within a hospital. - Regional: The intervention is implemented in an entire district, state, province or region, or in a group of institutions purposely sampled to represent a region. - National: The intervention is implemented in multiple districts/regions in a country, or the entire country, or in a group of institutions purposely sampled to represent a country. - Multinational: The intervention is implemented in multiple countries. | multiple-choice   \| 99 \| Cannot be determined \| \| --- \| --- \| \| 1 \| Individual \| \| 2 \| Unit-based or institutional \| \| 3 \| Regional \| \| 4 \| National \| \| 5 \| Multinational \| |
| **Summarise the primary research question(s) for the ITS analysis.**  Examples   - What are the effects of an antibiotic stewardship programme in a hospital aimed at reducing unnecessary antibiotic prescriptions, compared to no intervention? - What are the effects of a family planning intervention on pregnant women in intervention suburbs, compared to matched control suburbs? | text |
| **What is the country where the study was implemented?** | text |
| **Classify the country using the World Bank’s income group** | checkbox   \| 1 \| Low-income \| \| --- \| --- \| \| 2 \| Lower-middle income \| \| 3 \| Upper-middle income \| \| 4 \| High-income \| |
| **Information from the results reports** | |
| **Title of results report** | text |
| **What is the name of the publishing journal?** | text |
| **What is the source of funding?**  In-kind materials are also considered funding, and should be described in subsequent questions about the role of the funder. | checkbox   \| 99 \| Cannot be determined \| \| --- \| --- \| \| 0 \| No funding \| \| 1 \| Non-industry (non-profit, academic, government) \| \| 2 \| Industry \| \| 999 \| Other [elaborate] \| |
| **Briefly describe the intervention(s) or exposure(s) that constitute the interruption points.** | text |
| **At which level did the interruption occur, or was delivered / implemented?**   - Individual: The intervention is implemented in specific individuals without the intention to represent a geographical area. - Unit-based or institutions: The intervention is implemented in one or more specific institutions, hospitals or departments within a hospital. - Regional: The intervention is implemented in an entire district, state, province or region, or in a group of institutions purposely sampled to represent a region. - National: The intervention is implemented in multiple districts/regions in a country, or the entire country, or in a group of institutions purposely sampled to represent a country. - Multinational: The intervention is implemented in multiple countries. | multiple-choice   \| 99 \| Cannot be determined \| \| --- \| --- \| \| 1 \| Individual \| \| 2 \| Unit-based or institutional \| \| 3 \| Regional \| \| 4 \| National \| \| 5 \| Multinational \| |
| **At which level was the effect of the interruption measured in this study?**   - Individual: The intervention is implemented in specific individuals without the intention to represent a geographical area. - Unit-based or institutions: The intervention is implemented in one or more specific institutions, hospitals or departments within a hospital. - Regional: The intervention is implemented in an entire district, state, province or region, or in a group of institutions purposely sampled to represent a region. - National: The intervention is implemented in multiple districts/regions in a country, or the entire country, or in a group of institutions purposely sampled to represent a country. - Multinational: The intervention is implemented in multiple countries. | multiple-choice   \| 99 \| Cannot be determined \| \| --- \| --- \| \| 1 \| Individual \| \| 2 \| Unit-based or institutional \| \| 3 \| Regional \| \| 4 \| National \| \| 5 \| Multinational \| |
| **Summarise the primary research question(s) for the ITS analysis.**  Examples   - What are the effects of an antibiotic stewardship programme in a hospital aimed at reducing unnecessary antibiotic prescriptions, compared to no intervention? - What are the effects of a family planning intervention on pregnant women in intervention suburbs, compared to matched control suburbs? | text |
| **Is this an ITS study by design?**  The study needs to: (1) have the minimum of 3 data points for at least 2 segments; and (2) use a model that is consistent with ITS analysis methods (e.g. for a time series of monthly data, the model needs to have a continuous parameter that represents number of months before and after the interruption). | multiple-choice   \| 0 \| No \| \| --- \| --- \| \| 1 \| Yes \| |
| **Was the data collected retrospectively, prospectively, or both?**   - Retrospective: Data was already available or collected at the time of the submission of the protocol - Prospective: Data was collected after the submission of the protocol   Use your best judgment based on:  (1) the end date of the data collection period relative to the protocol’s submission date, or if not available  (2) the timing of the intervention, and  (3) authors” words. | checkbox   \| 99 \| Cannot be determined \| \| --- \| --- \| \| 1 \| Retrospective \| \| 2 \| Prospective \| |
| **Information from protocols – for each outcome** | |
| **Outcome name**  Use name reported by authors | text |
| **Is this a control outcome?** | multiple-choice   \| 1 \| Yes \| \| --- \| --- \| \| 0 \| No \| |
| **If the measurement of this outcome increases / becomes more positive, does this indicate a beneficial effect or a harmful effect?** | multiple-choice   \| 0 \| Harmful effect \| \| --- \| --- \| \| 1 \| Beneficial effect \| \| 99 \| Cannot be determined \| |
| **What is the type of matching of the outcome between the protocol to the results report?** | multiple-choice   \| 0 \| Not applicable (outcome missing in either protocol or results report) \| \| --- \| --- \| \| 1 \| One-to-one matching \| \| 2 \| One-to-many matching \| \| 99 \| Cannot be determined \| |
| **Was this outcome specified in the protocol?**  Examples   - Not as an ITS analysis: pre-post analysis, GEE models, DID analysis; - Unclear whether ITS analysis: authors provided no description of the model or equation for the analysis, or there was insufficient information to determine which outcome was analysed by ITS analysis; - Maybe: the protocol lists "neonatal outcomes" and the report lists "birth weight"; or the protocol lists "administration of painkillers" and the report lists "administration of oxycodone". | multiple-choice   \| 1 \| Yes, as an ITS analysis \| \| --- \| --- \| \| 2 \| Yes, but not as an ITS analysis \| \| 3 \| Yes, but unclear whether it is an ITS analysis \| \| 0 \| No, not specified at all \| |
| **Details about this outcome**  You can copy and paste info about this outcome from the text e.g. clinical definition, frequency, how the outcome will be measured and transformed | text |
| **Is this outcome defined as a primary or secondary outcome?**   - Primary outcome: (1) the authors explicitly labelled the outcome in the text as the “main” or “primary” outcome. Phrases like “primary analysis”, “main analysis”, “main objective” do not qualify as defining a outcome primacy. (2) the outcome was used in sample size or power calculation, or (3) the outcome is the only outcome in the study. - Secondary outcome: the authors explicitly labelled the outcome in the text as the “secondary” outcome. Specifying one outcome as a primary outcome does not automatically make the remaining outcomes secondary. - Could not be classified: the authors did not provide any information to the outcome into a primary or secondary outcome. | multiple-choice   \| 1 \| Primary \| \| --- \| --- \| \| 2 \| Secondary \| \| 99 \| Could not be classified \| |
| **What is the data type when measured at individual level?**  Consider how the data would have been recorded after measuring for each participant.  Examples   - Heart attack (occurred / not occurred) 🡪 binary; - Number of pills taken 🡪 count; - BMI score 🡪 continuous; - Patient satisfaction rating (very satisfied to very dissatisfied) 🡪 ordinal; - % of hospitalisation days without seizures 🡪 proportion. | checkbox   \| 99 \| Cannot be determined \| \| --- \| --- \| \| 1 \| Binary \| \| 2 \| Count \| \| 3 \| Continuous / ordinal \| \| 4 \| Proportion \| \| 999 \| Other [elaborate] \| |
| **What is the data type during the process of transforming data collected at the individual level for this study?**  This question is applicable when the raw patient data (e.g. from EHR records) undergoes any intermediate data processing steps for the purpose of this study.  If there are multiple steps of data transformation, check all that potentially apply.   - Cannot be determined: applicable when (1) you know there is some data processing involved, but unsure of how it was done; OR (2) data type at aggregation is also "Cannot be determined". - Not applicable: applicable when (1) you know the data type at aggregation AND (2) there is no intermediate data processing. | checkbox   \| 99 \| Cannot be determined \| \| --- \| --- \| \| 0 \| Not applicable \| \| 1 \| Binary \| \| 2 \| Count \| \| 3 \| Continuous / ordinal \| \| 4 \| Proportion \| \| 999 \| Other [elaborate] \| |
| **What is the data type that the individual data will be aggregated into for analysis?**  As the data is by default reported at frequent time intervals, descriptions referring to time intervals (monthly, weekly, etc.) do not automatically make the data a "rate". Instead, consider the data type at each time point.  Use information from: 1) description of the time series and the data being analysed; 2) graphs of the time series of the specific outcome.  Examples   - % of daily admissions that are attended to 🡪 proportion; - number of weekly heart attacks 🡪 count; - average monthly performance score 🡪 continuous; - incidence of preeclampsia per 10,000 women per hospital-day 🡪 rate. | multiple-choice   \| 99 \| Cannot be determined \| \| --- \| --- \| \| 1 \| Binary \| \| 2 \| Count \| \| 3 \| Proportion \| \| 4 \| Continuous / ordinal \| \| 5 \| Rate \| \| 999 \| Other [elaborate] \| |
| **Information from the results reports – for each outcome** | |
| **Outcome name**  Use name reported by authors | text |
| **Was this outcome specified in the results report**  **Examples**   - Not as an ITS analysis: pre-post analysis, GEE models, DID analysis; - Without any eligible results: outcome was mentioned but authors only provided ineligible results e.g. summary statistics from pre- and post-segments (without a measure of pre-post difference), graphs without trend lines; - Not specified at all: outcome was not mentioned at all. | multiple-choice   \| 1 \| Yes, with results from ITS analysis \| \| --- \| --- \| \| 2 \| Yes, with results from non-ITS analysis \| \| 3 \| Yes, mentioned but without any eligible results \| \| 0 \| No, not specified at all \| |
| **Details about this outcome**  You can copy and paste info about this outcome from the text e.g. clinical definition, frequency, how the outcome was measured and transformed | text |
| **Was this outcome defined as a primary or secondary outcome?**   - Primary outcome: (1) the authors explicitly labelled the outcome in the text as the “main” or “primary” outcome. Phrases like “primary analysis”, “main analysis”, “main objective” do not qualify as defining a outcome primacy. (2) the outcome was used in sample size or power calculation, or (3) the outcome was the only outcome in the study. - Secondary outcome: the authors explicitly labelled the outcome in the text as the “secondary” outcome. Specifying one outcome as a primary outcome does not automatically make the remaining outcomes secondary. - Could not be classified: the authors did not provide any information to the outcome into a primary or secondary outcome. | multiple-choice   \| 1 \| Primary \| \| --- \| --- \| \| 2 \| Secondary \| \| 99 \| Could not be classified \| |
| **What was the data type when measured at individual level?**  Consider how the data would have been recorded after measuring for each participant.  Examples   - Heart attack (occurred / not occurred) 🡪 binary - Number of pills taken 🡪 count - BMI score 🡪 continuous - Patient satisfaction rating (very satisfied to very dissatisfied) 🡪 ordinal - % of hospitalisation days without seizures 🡪 proportion | checkbox   \| 99 \| Cannot be determined \| \| --- \| --- \| \| 1 \| Binary \| \| 2 \| Count \| \| 3 \| Continuous / ordinal \| \| 4 \| Proportion \| \| 999 \| Other [elaborate] \| |
| **What was the data type during the process of transforming data collected at the individual level for this study?**  This question is applicable when the raw patient data (e.g. from EHR records) underwent any intermediate data processing steps for the purpose of this study.  If there are multiple steps of data transformation, check all that potentially apply.   - Cannot be determined: applicable when (1) you know there was some data processing involved, but unsure of how it was done; OR (2) data type at aggregation is also "Cannot be determined". - Not applicable: applicable when (1) you know the data type at aggregation AND (2) there was no intermediate data processing. | checkbox   \| 99 \| Cannot be determined \| \| --- \| --- \| \| 0 \| Not applicable \| \| 1 \| Binary \| \| 2 \| Count \| \| 3 \| Continuous / ordinal \| \| 4 \| Proportion \| \| 999 \| Other [elaborate] \| |
| **What was the data type that the individual data was aggregated into for analysis?**  As the data is by default reported at frequent time intervals, descriptions referring to time intervals (monthly, weekly, etc.) do not automatically make the data a "rate". Instead, consider the data type at each time point.  Use information from: 1) description of the time series and the data being analysed; 2) graphs of the time series of the specific outcome.  Examples   - % of daily admissions that are attended to 🡪 proportion - number of weekly heart attacks 🡪 count - average monthly performance score 🡪 continuous - incidence of preeclampsia per 10,000 women per hospital-day 🡪 rate | multiple-choice   \| 99 \| Cannot be determined \| \| --- \| --- \| \| 1 \| Binary \| \| 2 \| Count \| \| 3 \| Proportion \| \| 4 \| Continuous / ordinal \| \| 5 \| Rate \| \| 999 \| Other [elaborate] \| |
| **Did the authors provide justification for not reporting / reporting an unplanned outcome?**  Justification not needed: applicable for (1) control outcomes; (2) outcomes with one-to-many matches; (3) outcomes that were specified in both the protocol and results report. | multiple-choice   \| 1 \| Yes \| \| --- \| --- \| \| 0 \| No \| \| 99 \| Justification not needed \| |
| **Cite the justification provided by authors** | text |
| **Did the authors provide justification for the discrepancy in the primary / secondary outcome classification?**  Justification not needed: applicable for (1) control outcomes; (2) outcomes with one-to-many matches; (3) outcomes with no discrepancy. | multiple-choice   \| 1 \| Yes \| \| --- \| --- \| \| 0 \| No \| \| 99 \| Justification not needed \| |
| **Information from the results reports – for each result** | |
| **Did the authors report the estimate of this effect measure?**   - Unclear if this is an ITS analysis: author did not report any model or equation to confirm it was an ITS analysis - Unclear if this effect estimate is eligible: unclear whether the reported estimate can quantify the effect of the intervention e.g. unclear whether author is reporting a slope change (eligible) or a post-interruption slope (ineligible) - Not as an ITS analysis: any pre-post analysis that did not model a time parameter and only modelled a binary/categorical parameter for the different time segments e.g. DID analysis | multiple-choice   \| 99 \| Unclear if this is an ITS analysis \| \| --- \| --- \| \| 999 \| Unclear if this effect estimate is eligible \| \| 1 \| Yes, reported as an ITS analysis \| \| 2 \| Yes, reported but not as an ITS analysis \| \| 0 \| No, not reported at all \| |
| **Effect measure to be reported**  Only consider effect measures that quantify the impact of the interruption. Effect measures that are not of interest: pre-interruption level/slope; pre-post change in level/slope in the control series   - Other effect measures from ITS analysis: e.g. level at 12 months, intervention x year interaction term) - Other unidentified effect measures from non-ITS analysis: if it is clear that ITS was undertaken but unclear what the effect estimate is - Other effect measures from non-ITS analysis: e.g. odds ratios from simple regression, coefficients from difference-in-difference analysis | multiple-choice   \| 1 \| Level change from ITS analysis \| \| --- \| --- \| \| 2 \| Slope change from ITS analysis \| \| 3 \| Other known effect measures from ITS analysis [elaborate] \| \| 4 \| Other unidentified effect measures from ITS analysis [elaborate] \| \| 5 \| Other effect measures from non-ITS analysis [elaborate] \| \| 99 \| Unclear whether this effect estimate is ITS or not \| |
| **For this result, was there a comparison between an intervention and a control series?**   - No: the comparison was between pre- and post-interruption within the intervention series only - Yes: the comparison was between the pre-post change of the intervention series vs the pre-post change of the control series | multiple-choice   \| 99 \| Cannot be determined \| \| --- \| --- \| \| 0 \| No \| \| 1 \| Yes \| |
| **Magnitude of effect measure** | text |
| **Lower limit of the 95% confidence interval** | text |
| **Upper limit of the 95% confidence interval** | text |
| **Standard error** | text |
| **P-value e.g. 0.03, <0. 001** | text |
| **Qualitative statement about statistical significance, magnitude or direction of results**  e.g. “There is a significant increase in HIV testing rates after the intervention was rolled out”. | text |
| **Overall, is this a statistically significant result?**  "Yes" if p< 0.05 or declared statistically significant by the author in a qualitative statement.  A qualitative statement must mention “significant result" or "non-significant result". | multiple-choice   \| 99 \| Cannot be determined \| \| --- \| --- \| \| 1 \| Yes \| \| 0 \| No \| |
| **What is the direction of the effect measure?**  A qualitative statement must describe a trend (“no change”, “an increase/decrease in”), not just provide summary statistics pre- and post-. | multiple-choice   \| 99 \| cannot be determined \| \| --- \| --- \| \| 1 \| favouring interruption \| \| 0 \| favouring comparator / favouring neither side \| |
| **Where is the result located?** | text |

# **S4. Creating the database of ITS study protocols and their results reports**

**4.1. Creating a database of ITS study protocols**

**4.1.2. Literature search for protocols**

We searched eight bibliographic databases, five trial registries, four open-source repositories, two grey literature databases, one pre-print server and two open access journals that publish protocols. For MEDLINE, PubMed and Embase, we used a search filter designed to locate ITS studies with high sensitivity (16), and added keywords for protocols. The last search was on 12 January 2023, including all protocols from inception date until 31 December 2022.

**4.1.3 Screening of protocols**

One author (PYN) screened all titles and abstracts. A 10% random sample of abstracts deemed ineligible and all abstracts deemed eligible by PYN were independently screened by one of JEM, SLT, EK, or MJP. All full text articles were independently screened by two authors. Discrepancies were resolved through discussion between the screening authors or team discussions.

**4.1.4. Identifying the primary ITS research question(s)**

For each protocol, two authors (PYN and MJP) independently identified the “primary ITS research question(s)”; the ITS research question(s) reported by the authors as “primary”, or alternatively the first reported in the protocol. We used the primary ITS research question(s) to determine whether the study had been published and which outcomes and results were eligible for our assessment (see Section 5.2).

**4.2. Identifying corresponding results reports of ITS studies**

**4.2.1. Literature search for results reports**

For each protocol, we searched in PubMed, Ovid MEDLINE and Embase for corresponding results reports. The search strategy was tailored for each protocol, combining two elements: (a) identifiers of the study such as study’s name or acronyms, description of intervention, study registration number, etc. AND (b) either the first author, last author or the corresponding author. We additionally searched clinical trial registries (if applicable), Google Scholar and a forward citation tool. The initial search was conducted in January 2023 and three subsequent searches were conducted, once every 6 months, for all results reports published up to 30 June 2023.

**4.2.2. Screening for eligible results reports**

One author (PYN) screened the full text of all retrieved reports. 50% of full text reports deemed ineligible and 100% full text reports deemed eligible by PYN were independently screened by one of JEM/SLT/EK/MJP.

During screening, we first checked that the result report addressed the primary ITS research question(s) that we had identified in its corresponding protocol. In determining this, we considered the population/setting, interruption group(s) and the comparator group(s) elements of the research question(s). In addition, we checked whether the report cited the original protocol, or could be linked to the protocol via details such as trial registration number. If we were unsure, we contacted the corresponding authors to clarify. If the authors did not respond, a decision was reached via team discussion.

# **S5. Key definitions and eligibility criteria used in screening**

# **Eligible protocols of ITS studies**

Eligible protocols include protocols and statistical analysis plans of ITS studies.

We defined an ITS study based on the following criteria:

1. Design of the time series: The study involved a time series with the following features: (i) there were at least two segments separated by a clearly defined interruption (i.e. an intervention or an exposure), (ii) there were at least three data points for at least two of the segments, and (iii) each data point represented a summary statistic (e.g., mean or rate) of individual observations collected from a group of individuals (e.g., within a country, state, hospital, or other unit) within a period of time (e.g., weekly or monthly);
2. Intention to undertake an ITS analysis: Indication of such an intention includes: (a) specifying “interrupted time series” in the title, abstract or the Methods section of the article, or (b) describing statistical methods consistent with ITS analysis methods, such as segmented regression, or an autoregressive integrated moving average (ARIMA) model in the presence of an interruption in the time series.

If the design criteria for the time series were met but the authors only planned to undertake non-ITS analyses (e.g., simply comparing the pre- and post-interruption means without modelling time trend), the protocols were excluded. Alternatively, if the authors expressed an intention to undertake an ITS analysis but the time series failed any of the design criteria (e.g. having fewer than three data points for one segment), the protocols were also excluded. If there was insufficient information about the design of the time series, we only assessed criterion (b), with studies meeting this criterion being included.

Studies that planned to conduct ITS analysis alongside other types of analyses (e.g., qualitative analysis or cost-effectiveness modelling) were eligible. Both controlled and uncontrolled ITS studies were eligible. Studies that used the ITS design to examine the effects of an intervention on individuals (e.g. using multilevel model with a random slope term for time at the participant level) were ineligible. We excluded conference abstracts and protocols not written in English.

# **Primary ITS research questions**

For each protocol, we identified the “primary ITS research question”, defined as the ITS research question that was reported by the authors as the “primary analysis”, “primary objective” or “primary research aim”; or alternatively, the first research question specified under the Research Aims/Objectives or Methods section that was planned to be analysed using ITS analysis methods. If a protocol included both an ITS analysis and other types of analyses (e.g., qualitative analysis or cost-effectiveness modelling), we only considered the ITS analysis for the primary ITS research question.

To form the primary ITS research question, we extracted information about the following elements: the population/setting (P), the interruption group(s) (I), and the comparator group(s) (C). For the purpose of our study, we use the term “group” to refer to interventions or exposures that occur in different time periods or segments. If there were multiple interventions investigated, the first mentioned intervention was selected for primary ITS research question. We did not include the outcome elements (O) in the primary ITS research question, because our interest lay in examining outcome/result reporting bias. The primary ITS research question did not have to include all of the abovementioned elements.

We constructed the primary ITS research question using the reported elements: *What is the effect of [intervention], implemented at [setting / location], compared to [comparator periods / comparison sites / comparison group]?*

Case study: Suppose an ITS has three segments. The first segment is the pre-implementation period; the second is the implementation period and the third is the post-implementation period. The intervention is evaluated in two populations (population 1 and 2).


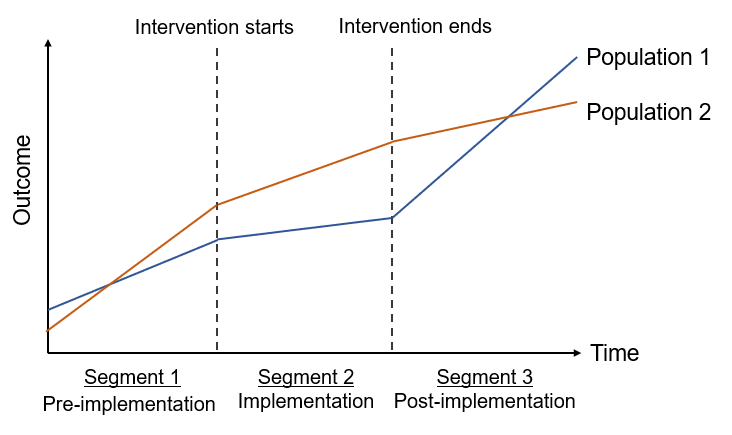


If the authors stated “Our primary aim is to examine the effect of the implementation period and that of the post-implementation periods in population 1”, then we constructed the primary ITS research questions to be:

- What is the effect of implementation period in population 1 compared with pre-implementation?
- What is the effect of post-implementation period in population 1 compared with pre-implementation?
- What is the effect of post-implementation period in population 1 compared with implementation?

If the authors stated “Our primary aim is to investigate the change in outcome following the commencement of the intervention”, we would combine the implementation and post-implementation periods as one segment, and consider any results pertaining to both population 1 and 2 (since the population was not stated in the aim). We constructed the primary ITS research questions to be:

- What is the effect of the implementation and post-implementation periods in population 1, compared with pre-implementation?
- What is the effect of the implementation and post-implementation periods in population 2, compared with pre-implementation?

# **Eligible reports of ITS results**

Report(s) of the results were defined as any peer-reviewed report that met the following criteria:

1. The report addressed the same primary ITS research question(s) as the protocol; AND
2. The report either (1) acknowledged and cited the original protocol, or (2) matched the original protocol in at least one of the following details: funding or grant number, ethics application number, trial registration number, unique name or acronyms of the intervention.

When multiple eligible results reports were found, we included all of them. We included results reports regardless of the outcomes specified in the protocol. For example, if the authors stated in the protocol that they aimed to evaluate an intervention designed to reduce cardiovascular adverse outcomes among hypertension patients, when screening the potential results report, we checked if the intervention was designed to reduce cardiovascular adverse outcomes, but we did not exclude a results report if they only reported other outcomes (e.g., quality of life) that were not specified in the protocol.

Exclusion criteria: We excluded conference abstracts and short reports, and reports not written in English. Methods papers in which data from the ITS was used, for example, to demonstrate the impact of using different statistical analysis methods, were ineligible.

If we were uncertain whether a report was indeed the results of research carried out under a protocol (e.g., when the primary ITS research question was the same but there was no citation of the protocol nor any of the abovementioned detail), we contacted the corresponding author of the protocol to clarify. If the author did not respond after two weeks, the team discussed and reached a consensus on the eligibility of the results report.

# **Eligible outcomes**

In this study, an outcome is defined by the combination of the following five elements: domain, specific measure, time points, data type of individual measurements, and data type at aggregation (Table 1). Outcomes were considered distinct if they differed in any of the elements.

For each ITS study, we extracted all outcomes pertaining to the primary ITS research question(s), with outcomes extracted separately from the protocol and from the results report.

# **Principles in matching an outcome in the protocol with a corresponding outcome in the results report**

- Each outcome in the protocol was assessed for a match with outcomes extracted from the results report.
- An outcome’s description did not have to have all five elements. The authors might only provide a description for one element, fewer than five elements, or all five elements.
- When comparing an outcome in the protocol with an outcome in the results report, we compared the elements that were presented in the outcome description in both the protocol and the results report (which we term ‘common elements’).
- If the two outcomes were matching in all of their common elements, they were classified as ‘matched’ i.e. the outcome was reported in both the protocol and the results report (see examples 1, 3-4 in section 5.4.2).
- If two potential outcomes differed in any of the common elements, they were classified as ‘not matching’ (see examples 2, 5-6 in section 5.4.2).
- The two outcomes might have different elements in their descriptions and still be matched, as long as the common elements were matched (see examples 3-4 in section 5.4.2)
- One outcome in protocol could be matched to one outcome (one-to-one matching) or multiple outcomes (one-to-many matching) in the results report, or none at all (see section 5.4.3).
- If an outcome in the protocol could be matched to a single outcome in the results report (one-to-one matching), the outcome was considered as reported in both documents. The outcome in both documents was then assessed for any further discrepancy in outcome primacy.
- If an outcome in the protocol did not have a match, the outcome was considered as planned in the protocol and omitted in the results report, which constituted a discrepancy in outcome reporting.
- If an outcome in the results report did not have a match, the outcome was considered as unplanned in the protocol and added in the results report, which constituted a discrepancy in outcome reporting.
- If an outcome in the protocol could be matched to multiple outcomes in the results report (one-to-many matching), the outcome was not assessed for discrepancy.

# **Examples of applying the principles in matching two outcomes**

Assume an outcome that can be fully described as follows:

| **Systolic blood pressure (SBP)** *[domain]*, measured in **mmHg** *[data type of individual measurements (continuous)]* using a **sphygmomanometer** *[specific measure]*, at **24 hours** *[time point]* after intervention. The monthly **proportion of patients** *[data type at aggregation (proportion)]* with SBP over 180mmHg will analysed using segmented linear regression. |
| --- |

| **Eg** | **Outcome’s description in the protocol** | **Outcome’s description(s) in the results report** | **Result of matching** |
| --- | --- | --- | --- |
| 1 | SBP in mmHg will be measured at 24 hours after intervention using a sphygmomanometer. The monthly mean SBP of all patients in the hospital will be analysed using segmented linear regression. | SBP in mmHg was measured at 24 hours after intervention using a sphygmomanometer. The mean SBP of all patients in the hospital was aggregated monthly into a time series for analysis. | Same number of elements, all elements matched 🡪 matched |
| 2 | SBP in mmHg will be measured at 24 hours after intervention using a sphygmomanometer. All patient SBP measurements will be averaged monthly to construct a time series of monthly mean SBP. | SBP was measured using a sphygmomanometer. The SBP (mmHg) was measured at 48 hours after IV administration for all patients and the monthly mean SBP was aggregated into a time series. | Same number of elements, some elements mismatched (monthly vs weekly) 🡪 not matched |
| 3 | SBP will be measured at 24 hours after intervention. | SBP was measured at 24 hours after intervention and the monthly mean SBP of all 300 patients in the hospital was aggregated into a time series. | Different number of elements, all common elements matching 🡪 matched |
| 4 | SBP will be measured at 24 hours after intervention and the monthly mean SBP of all patients in the hospital will be aggregated into a time series. | SBP was measured at 24 hours after intervention. | Different number of elements, all common elements matching 🡪 matched |
| 5 | We will analyse the effect of the intervention using a time series of mean SBP among all patients admitted to the hospital each month. All SBP measurements will be taken 24 hours after intervention. | SBP was measured 24 hours after intervention and a time series of proportion of patients with SBP>200mmHg was constructed. | Different number of elements, some common elements mismatched 🡪 not matched |
| 6 | A time series of monthly proportion of patients with SBP >180mmHg (SBP measured 24 hours after intervention) will be constructed. | We constructed a time series of monthly proportion of patients with SBP >200mmHg (SBP measured 24 hours after intervention using a sphygmomanometer) | Different number of elements, some common elements mismatched 🡪 not matched |

*Any shaded phrase denotes an element in the outcome definition framework. A blue shaded phrase denotes a common element that has matching detail between the protocol and the results report. An orange shaded phrase denotes a common element that has mismatched detail between the protocol and the results report. A grey shaded phrase denotes an element that is only present in either the protocol or the results report and is not compared.*

# **Examples on matching outcomes from the protocol with outcomes in the results report**

| **Eg** | **Outcome’s description  in the protocol** | **Outcome’s description(s)  in the results report** | **Result of matching** | **Type of matching** |
| --- | --- | --- | --- | --- |
| 1 | - Sale volume for vegetables, meat and confectionery | - Sale volume for vegetable and confectionery | - Sale volume (vegetable) 🡪 Sale volume (vegetable) - Sale volume (confectionery) 🡪 Sale volume (confectionery) - Sale volume (meat) 🡪 (X) | - One-to-one - One-to-one - No match |
| 2 | - Depression score | - Depression severity, measured by the HADS-D and BDI scores | - Depression 🡪 HADS-D - Depression 🡪 BDI | - One-to-many - One-to-many |
| 3 | - HADS-D score | - Depression severity, measured by the HADS-D and BDI scores | - HADS-D 🡪 HADS-D - (X) 🡪 BDI | - One-to-one - No match |
| 4 | - Mean FIM score | - Mean FIM score - Number of patients with ≥22 points change in FIM score | - Mean FIM score 🡪 Mean FIM score - (X) 🡪 Number of patients with ≥22 points change in FIM score | - One-to-one - No match |
| 5 | - Number of rotarovirus admissions, based on ICD-10 diagnosis | - Number of rotarovirus admissions, based on laboratory confirmation | - Number of rotarovirus admissions (ICD-10) 🡪 (X) - (X) 🡪 Number of rotarovirus admissions (laboratory) | - No match - No match |
| 6 | - Composite outcome (cardiovascular death and MI) | - Composite outcome (cardiovascular death, MI, stroke) | - Composite outcome (cardiovascular death and MI) 🡪 (X) - (X) 🡪 Composite outcome (cardiovascular death, MI, stroke) | - No match - No match |
| 7 | - Inappropriate indications for IV catheters | - Inappropriate indications for central IV catheters - Inappropriate indications for peripheral IV catheters - Inappropriate indications for urethral catheters | - Inappropriate indications for IV catheters 🡪 Inappropriate indications for central IV catheters - Inappropriate indications for IV catheters 🡪 Inappropriate indications for peripheral IV catheters - (X) 🡪 Inappropriate indications for urethral catheters | - One-to-many - One-to-many - No match |

*(A) 🡪 (B) denotes that outcome (A) in the protocol was matched to outcome (B) in the results report.*

*(X) 🡪 (B) denotes that outcome (B) in the report of result had no match in the protocol i.e. the outcome was not specified in the protocol.*

*(A) 🡪 (X) denotes that outcome (A) in the protocol had no match in the results report i.e. the outcome was not specified in the results report.*

*Abbreviations: BDI: Beck Depression Inventory; FIM: Functional Independence Measure; HADS-D: Hospital Anxiety and Depression Scale – Depression subscale; IV: intravenous; MI: myocardial infarction.*

# **Examples of one-to-many matches (i.e. one outcome in the protocol matched to multiple outcomes in the results report)**

| **Eg** | **Outcome’s description in the protocol** | **Outcome’s description(s) in the results report** | **Notes** |
| --- | --- | --- | --- |
| 1 | - health outcomes | - number of cases of postnatal depression - number of cases of postpartum haemorrhage | The protocol only provided a broad domain of the outcome, without specifying the list of health outcomes that were to be investigated. |
| 2 | - process evaluation outcomes | - number of referrals from primary providers - number of prescriptions dispensed - number of hospital transfers | The protocol only provided a broad domain of the outcome, without specifying the list of process evaluation outcomes that were to be investigated. |
| 3 | - rate of prescription of antibiotics | - rate of prescription of co-amoxiclav - rate of prescription of ciprofloxacin - rate of prescription of levofloxacin | The protocol did not specify which antibiotics were to be investigated. |
| 4 | - sale of opioids | - sale of oxycodone - sale of codeine - sale of fentanyl - sale of any opioid | The protocol did not specify which opioid were to be investigated. |
| 5 | - catheter-related complications | - extravasation - haematuria - dislocation of catheters | The protocol did not specify which catheter-related complications were to be investigated. |
| 6 | - inappropriate indications for intravenous catheters | - inappropriate indications for catheters – central venous catheters - inappropriate indications for catheters – peripheral intravenous catheters | The protocol did not specify that there were different types of intravenous catheters, and did not specify which type of intravenous catheters were to be investigated. |
| 7 | - substance use | - drinking - illicit drug use - any drinking or illicit drug use | The protocol did not specify which types of substance use were to be investigated. |

# **Examples of discrepancies in outcome reporting**

| **Eg** | **Protocol** | **Results report** | **Decision** |
| --- | --- | --- | --- |
| 1 | Outcome A was specified as primary outcome.  Outcomes B and C were specified as secondary outcomes. | Eligible results (e.g. slope changes) were reported for outcomes A, B and C.  Outcome A was specified as primary outcome.  Outcomes B and C were specified as secondary outcomes. | Outcome A: no discrepancy  Outcome B: no discrepancy  Outcome C: no discrepancy |
| 2 | Outcome A was specified as primary outcome.  Outcomes B and C were specified as secondary outcomes. | Eligible results (e.g. slope changes) were reported for outcomes A, B and C.  Outcome A was specified as primary outcome.  No mention of outcome primacy for outcomes B and C. | Outcome A: no discrepancy  Outcome B: no discrepancy  Outcome C: no discrepancy |
| 3 | Outcomes A and B were specified as primary outcomes. | Eligible results were reported for outcomes A, B and C.  Outcome A was used in power calculation.  No mention of outcome primacy for all outcomes. | Outcome A: no discrepancy (as outcome A was used in power calculation and thus classified as the primary outcome)  Outcome B: discrepancy – primary outcome demoted to unclassified outcome  Outcome C: discrepancy – missing in protocol |
| 4 | Outcome A was specified.  No mention of outcome primacy. | An eligible result was reported for outcome A.  No mention of outcome primacy. | Outcome A: no discrepancy  (as outcome A was the only outcome and classified as the primary outcome) |
| 5 | Outcomes A, B, C were specified.  No mention of outcome primacy for all outcomes. | Eligible results were reported for outcomes A, B and C.  Outcome A was specified as primary outcome.  Outcomes B and C were specified as secondary outcomes. | Outcome A: discrepancy – unclassified outcome promoted to primary outcome.  Outcomes B and C: no discrepancy |
| 6 | Outcomes A, B, C were specified.  No mention of outcome primacy for all outcomes. | Eligible results were reported for outcomes C and D.  Outcome B was mentioned without any eligible result.  No mention of outcome primacy for all outcomes. | Outcome A: discrepancy – missing in results report  Outcome B: discrepancy – missing in results report  Outcome C: no discrepancy  Outcome D: discrepancy – missing in protocol |

# **Eligible results**

We defined an “eligible result” as any measure of a difference between the two segments of interest; for example, the difference between the pre-intervention segment and post-intervention segment. An eligible result could be either (1) a numerical result: an effect estimate with/without the 95% confidence interval or standard error, or a P value; or (2) a qualitative statement about the change between two time segments (e.g., “There is a statistically significant increase in the level of [outcome] between the two time periods”). Presentation of only summary statistics within each period (e.g., means) were ineligible.

An eligible result could be from an analysis that was or was not an ITS analysis (Section 5.1 for further details on what was considered an ITS analysis) or for an outcome that was not specified in the protocol, as long as it addressed the primary ITS research question(s). However, if the result corresponded to non-ITS analyses that were pre-specified in the ITS study protocol, it was ineligible.

# **S6. Discrepancies in outcome reporting | Sensitivity analysis: justified discrepancies were classified as no discrepancy**

| **Type of discrepancies** | **Number of outcomes (%) (N=388)** | **Number of outcomes per study, median (IQR) (44 studies)** | **Percentage of outcomes per study, median (IQR) (44 studies)** |
| --- | --- | --- | --- |
| No discrepancy | 242 (62%) | 2 (0 to 6) | 56% (9% to 100%) |
| Discrepancy | 146 (38%) | 2 (0 to 5) | 20% (0% to 55%) |
| Missing in protocol | 48 (12%) | 0 (0 to 0) | 0% (0% to 0%) |
| Missing in results report | 83 (21%) | 0 (0 to 2) | 1% (0% to 34%) |
| Discrepancy in outcome primary | 15 (4%) | 0 (0 to 0) | 0% (0% to 0%) |
| Primary outcome was demoted to secondary/unclassified outcome | 4 (1%) | 0 (0 to 0) | 0% (0% to 0%) |
| Secondary/unclassified outcome was promoted to primary outcome | 11 (3%) | 0 (0 to 0) | 0% (0% to 0%) |

*In this sensitivity analysis, 56 outcomes for which authors provided a justification for discrepancies were re-classified from “discrepancy” to “no discrepancy”.*

*^†^Each outcome can have multiple results. For example, after fitting a segmented linear regression, the authors can report two results for each outcome: an estimate of the slope change and an estimate of the immediate level change.*

*Abbreviation: IQR: interquartile range; ITS: interrupted time series; RR: results report*

# **S7. Results from marginal logistic regression**

| **Variable** | **Number of results**^*^ | **Odds ratio (95% confidence interval)** | **P value** |
| --- | --- | --- | --- |
| Favourable status  (1=Favourable to the interruption;  0=Not favourable to the interruption) | 842 | 1.06 (0.74 to 1.53) | 0.746 |
| Funding  (1=Industry funding;  0=No funding or non-industry funding) | 842 | 1.50 (0.21 to 10.43) | 0.685 |
| Outcome primacy^†^ (1=Primary outcome;  0=Secondary or unclassified outcome) | 842 | 1.77 (0.75 to 4.18) | 0.195 |

*^*^18 results were excluded from the regression because their favourable status could not be determined.*

^†^*Outcome primacy was* *recorded based on what was reported in the results report, or if not available, in the protocol. If outcome primary was not specified in both the results report and the protocol, it was treated as “unclassified” for this analysis.*

*This analysis used a marginal logistic regression model with generalised estimating equations, an exchangeable working correlation, robust standard errors clustered by study, and fixed effects for: whether a result is favourable to the interruption or not, and potential confounders: type of funding (industry, non-industry [government, education institutions or not-for-profit entities] or no funding [as declared by authors]) and outcome primacy (primary or secondary / unclassified)*
